# Genetically programmed cell-based synthesis of non-natural peptide and depsipeptide macrocycles

**DOI:** 10.1038/s41557-022-01082-0

**Published:** 2022-12-22

**Authors:** Martin Spinck, Carlos Piedrafita, Wesley E. Robertson, Thomas S. Elliott, Daniele Cervettini, Daniel de la Torre, Jason W. Chin

**Affiliations:** Medical Research Council Laboratory of Molecular Biology, Cambridge, UK

**Keywords:** Synthetic biology, Biosynthesis, Peptides

## Abstract

The direct genetically encoded cell-based synthesis of non-natural peptide and depsipeptide macrocycles is an outstanding challenge. Here we programme the encoded synthesis of 25 diverse non-natural macrocyclic peptides, each containing two non-canonical amino acids, in Syn61Δ3-derived cells; these cells contain a synthetic *Escherichia coli* genome in which the annotated occurrences of two sense codons and a stop codon, and the cognate transfer RNAs (tRNAs) and release factor that normally decode these codons, have been removed. We further demonstrate that pyrrolysyl-tRNA synthetase/tRNA pairs from distinct classes can be engineered to direct the co-translational incorporation of diverse alpha hydroxy acids, with both aliphatic and aromatic side chains. We define 49 engineered mutually orthogonal pairs that recognize distinct non-canonical amino acids or alpha hydroxy acids and decode distinct codons. Finally, we combine our advances to programme Syn61Δ3-derived cells for the encoded synthesis of 12 diverse non-natural depsipeptide macrocycles, which contain two non-canonical side chains and either one or two ester bonds.

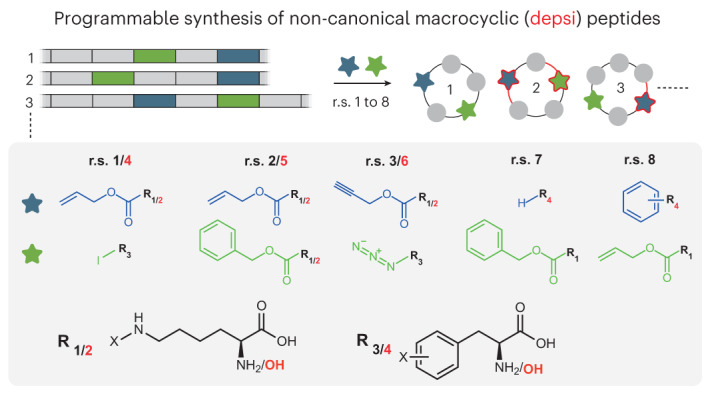

## Abstract (web summary)

Macrocyclic peptides can be genetically encoded and synthesized in cells; however, the programmable diversity is limited. Now, macrocycles containing two non-canonical amino acids have been genetically encoded and synthesized in codon-reassigned Syn61Δ3 cells. Incorporating diverse hydroxy acids in Syn61Δ3 cells enables the synthesis of non-natural depsipeptides containing either one or two ester bonds.

## Main

Nature synthesizes peptide and depsipeptide macrocycles—including many antibiotics, immunosuppressants and antitumor compounds—that contain an array of non-canonical monomers. These molecules are synthesized using either: (1) megadalton protein complexes (non-ribosomal peptide synthetases) or (2) post-translational modification of the canonical amino acids after ribosomal polymerization (for example, ribosomally synthesized and post translationally modified peptides, RiPPs). Despite substantial efforts over several decades, and notable successes^1–6^, the ability to engineer these systems to predictably generate any desired product remains largely elusive. Thus, reliably programming the cellular synthesis of macrocycles with non-canonical side chains and backbones remains an outstanding challenge.

In vitro translation is used to encode diverse macrocycles^7^, and proteins and peptides incorporating alpha hydroxy acids^8,9^. However, we are not aware of examples of macrocyclic depsipeptide synthesis via in vitro translation. The cellular synthesis of macrocyclic peptides composed of the canonical amino acids has been realized^10^, but progress on extending these approaches to produce non-canonical macrocycles has been limited to encoding one non-canonical amino acid (ncAA), with limited efficiency, in response to the amber stop codon^11^. Notably, the creation of macrocyclic depsipeptides (or any non-alpha-l-amino acid linked macrocycle) via cell-based translation, has not—to the best of our knowledge—been addressed.

We previously compressed the genetic code of *Escherichia coli* through the design and total synthesis of a functional recoded *E. coli* genome^12^. In the synthetic genome we systematically replaced annotated occurrences of two sense codons (TCG and TCA, which canonically encode serine) with defined synonyms, and we also replaced the amber stop codon (TAG) with the ochre stop codon (TAA). The resulting cell (named Syn61) used 61 codons, rather than the canonical 64 codons, to encode cellular protein synthesis. After further directed evolution of Syn61, we deleted the transfer RNAs (tRNAs) that decode the TCG and TCA codons and the release factor (RF-1) that terminates protein synthesis in response to the amber stop codon; we thus created Syn61Δ3 and its evolved derivatives, which included Syn61Δ3(ev5), in which two tRNAs and RF-1 were deleted. Finally, we showed that we could introduce the codons no longer present in the genome into synthetic genes and reassign these codons, using mutually orthogonal engineered aminoacyl-tRNA synthetase (aaRS)/tRNA pairs, to ncAAs; this enabled the synthesis of proteins that contained several ncAAs, the encoded cellular synthesis of polymers composed entirely of ncAAs and the encoded synthesis of a single five-membered macrocycle that contained ncAAs^13^.

Here we combine ten synthetic genes and three distinct codon reassignment schemes in Syn61Δ3-derived cells to programme the cell-based synthesis of diverse non-natural macrocyclic peptides, each of which contains two ncAAs. We further demonstrate that pyrrolysyl-tRNA synthetase/tRNA pairs from distinct classes can be engineered to direct the co-translational incorporation of diverse alpha hydroxy acids, with both aliphatic and aromatic side chains. We define 77 engineered mutually orthogonal pairs that recognize distinct ncAAs or alpha hydroxy acids and decode distinct codons, and thus generate 49 reassignment schemes for encoding combinations of amino acids and hydroxy acids or for encoding two distinct hydroxy acids. Finally, we combine our advances to programme Syn61Δ3-derived cells for the encoded synthesis of 12 diverse non-natural depsipeptides; each depsipeptide contains two non-canonical side chains and either one or two ester bonds.

## Results

### Combinatorially encoding ncAAs in macrocyclic peptides

**Fig. 1:**
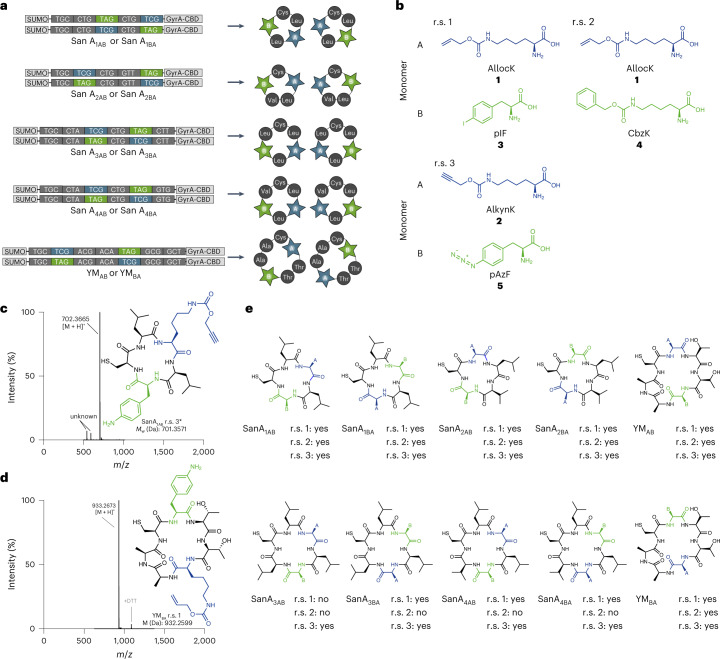
Genetically programmed cell-based synthesis of non-natural macrocycles. **a**, Macrocycles and the genes that programme their cell-based synthesis; macrocycle sequences are inspired by the natural sequences of SanA_1_–SanA_4_ and YM (Supplementary Fig. 1). The TCG codon (blue) programmes the incorporation of non-canonical monomer A (blue star), and the TAG codon (green) programmes the incorporation of non-canonical monomer B (green star). Macrocycle sequences are expressed as SUMO–intein (gyrA) fusions in Syn61∆3(ev5) and cyclized by the addition of Ulp1 and DTT^13^. Leu, Val, Cys, Ala and Thr (grey spheres) are canonical amino acids. **b**, r.s. 1–3 used for macrocyclic peptide synthesis. The reassignment schemes define the identity of monomers A and B; monomer A (blue) is incorporated in response to the TCG codon and monomer B (green) is incorporated in response to the TAG codon. **c**,**d**, Mass spectra and structures of purified SanA_1BA_ r.s. 3 (**c**) and YM_BA_ r.s. 1 (**d**). The asterisk denotes that the azide of pAzF is reduced to an amine, as previously reported^34^. The sequence synthesized is defined by the macrocycle sequence (defined in **a**) and the reassignment scheme (defined in **b**); thus, SanA_1BA_ r.s. 3 defines the SanA_1BA_ sequence in which the identity of A and B are defined by r.s. 3. **e**, Chemical structure of all the cyclic peptides, positions of ncAAs A and B are indicated. For each r.s. 1–3, ‘yes’ indicates that we detected the exact mass of the peptide after its purification, and ‘no’ indicates that we could not detect the peptide; see Supplementary Fig. 2 for the raw spectra and structure of the confirmed cyclic peptides. We were unable to express the fusion of SanA_3AB_ or SanA_3BA_ with r.s. 2, and we were unable to extract the peptides generated from SanA_4AB_ or SanA_4BA_ using r.s. 2 or from SanA_3AB_ with r.s. 1. The cyclic peptide precursors were purified by Ni–NTA (nitrilotriacetic acid) and the peptide excised using Ulp1 and DTT.

We designed ten genes encoding sequences inspired by the cyclic depsipeptides Sansalvamide A^14^ and YM-254890^15^ (Fig. 1a and Supplementary Fig. 1). We placed TCG and TAG codons within the macrocycle-encoding genes to direct the incorporation of non-canonical monomers. To enable excision and cyclization to generate the desired macrocycles, we encoded the genes as fusions to an N-terminal SUMO and a C-terminal GyrA intein-CBD domain^13^.

We encoded ncAAs (**1**–**5**) in response to the TCG and TAG codons in the macrocycle-encoding genes according to reassignment schemes 1–3 (r.s. 1–3). In each recoding scheme, monomer A is encoded in response to TCG and monomer B is encoded in response to TAG (Fig. 1a,b). The ncAAs introduced within each reassignment scheme were directed into the peptide using engineered mutually orthogonal aaRS/tRNA pairs; each aaRS/tRNA pair recognizes one ncAA and decodes either the TCG or TAG codon. In r.s. 1, monomer A is N_ε_-Alloc-l-lysine (**1**, AllocK) and is incorporated using the *Methanosarcina mazei* (*Mm*)PylRS/*Mm*tRNA^Pyl^_CGA_ pair^16^, and monomer B is *p*-iodophenylalanine (**3**, pIF) and is incorporated using the *Archeoglobus fulgidus* (*Af*)TyrRS(pIF)/*Af*tRNA^Tyr(A01)^_CUA_ pair^17^. In r.s. 2, monomer A is AllocK, and is incorporated using the *Mm*PylRS/*Mm*tRNA^Pyl^_CGA_ pair, and monomer B is N_6_-carbobenzyloxy-l-lysine (**4**, CbzK), and is incorporated using the *Methanomethylophilus* sp. *1R26* (*1R26*)PylRS(CbzK)/*Alv*tRNA^ΔNPyl(8)^_CUA_ pair^18^. In r.s. 3, monomer A is N_ε_-((prop-2-yn-1-yloxy)carbonyl)-l-lysine (**2**, AlkynK), and is incorporated using the *Mm*PylRS/*Mm*tRNA^Pyl^_CGA_ pair, and monomer B is *p*-azido-L-phenylalanine (**5**, pAzF) and is incorporated using the *Af*TyrRS(pAzF)/*Af*tRNA^Tyr(A01)^_CUA_ pair^17^.

We aimed to programme the synthesis of 30 diverse non-natural macrocycles by combining the 10 macrocycle-encoding fusion genes and the 3 ncAA reassignment schemes. For each combination tested, we aimed to express and purify the resulting fusion proteins from Syn61Δ3(ev5), and then to excise and cyclize the peptide, with Ulp1 and 1,4-dithiothreitol (DTT), in vitro. The resulting peptides were extracted after 16 hours of incubation by chloroform/isopropanol extraction. For 25 of the combinations tested, we produced the desired cyclic peptide, as judged by electrospray ionization mass spectrometry (ESI–MS) (Fig. 1c–e and Supplementary Fig. 2). Our results demonstrate that we can programme the combinatorial cell-based synthesis of diverse ncAA-containing macrocycles. Within each macrocycle, the position of each ncAA is defined by the genetic sequence, and the identity of the ncAA at each position is defined by the reassignment scheme.

### Encoding aliphatic hydroxy acids with PylRS/tRNA pairs

**Fig. 2:**
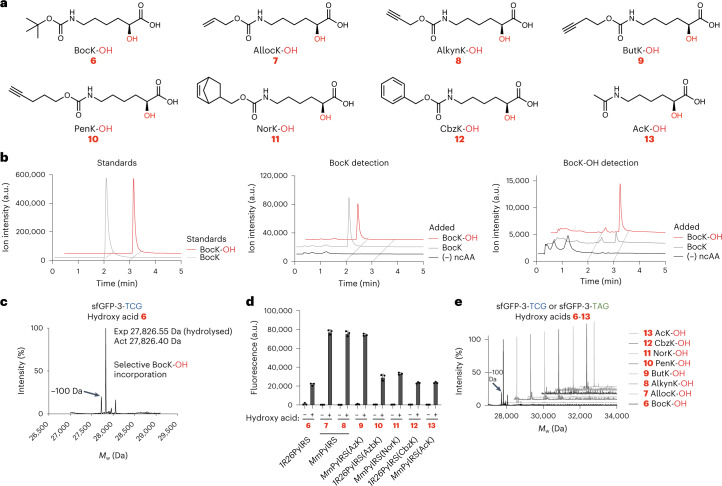
Genetically encoding hydroxy acids in response to TAG and TCG codons in Syn61∆3(ev5). **a**, Structures of the ‘aliphatic’ hydroxy acids **6**–**13**. **b**, Intracellular detection of **6** and its amino acid counterpart (BocK). Left: standards (100 µM) were used to define the retention times of BocK and **6**. The minor peak at 2 min detected with the **6** channel corresponds to the ^13^C isotope of BocK, which has the same mass as **6**. After the incubation of DH10B cells with the relevant ‘added’ compound (2 mM **6** (red) or 2 mM BocK (grey)), extracts were subjected to LC–MS assays, with selected ion monitoring, to detect BocK (middle) or **6** (right). In cells with BocK added (grey), only BocK was detected. In cells with **6** added (red), both **6** and BocK were detected. **c**, The production of sfGFP-His_6_ incorporating hydroxy acid **6**, sfGFP(3BocK-OH)-His_6_, from a sfGFP-3-TCG gene in Syn61∆3(ev5) cells harbouring the *Mm*PylRS/*Mm*tRNA^Pyl^_CGA_ orthogonal pair was confirmed by ESI–MS—only a mass that corresponds to hydroxy acid incorporation followed by ester bond cleavage at position 3 was detected. The minor peak at −100 Da results from loss of *tert*-butoxycarbonyl from **6**. **d**, Orthogonal PylRS active-site variants direct the incorporation of hydroxy acids **6**–**13** into sfGFP. All the *Mm*PylRS variants were paired with *Mm*tRNA^Pyl^_CGA_ to decode TCG, and all the *1R26*PylRS variants were paired with *Alv*tRNA^ΔNPyl(8)^_CUA_ to decode TAG (the data show sfGFP fluorescence in the absence (–) or presence of hydroxy acids). Error bars represent standard deviation from the mean of three biological replicates. PylRS active-site mutations^18,19,35,36^ are provided in Supplementary Table 2. **e**, Production of sfGFP-His_6_ incorporating hydroxy acids **6**–**13**, as confirmed by ESI–MS. Expressions were performed in Syn61∆3(ev5) cells supplemented with a hydroxy acid and its cognate PylRS/tRNA pair. **6** (hydrolysed): expected mass (exp) 27,826.26 Da, actual mass (act) 27,826.40 Da. **7** (hydrolysed): exp 27,810.23 Da, act 27,809.80 Da. **8** (hydrolysed): exp 27,808.20 Da, act 27,809.60 Da. **9** (hydrolysed): exp 27,822.26 Da, act 27,822.50 Da. **10** (hydrolysed): exp 27,836.29 Da, act 27,836.00 Da. **11** (hydrolysed): exp 27,876.32 Da, act 27,876.60 Da. **12** (hydrolysed): exp 27,860.28 Da, act 27,860.20 Da. **13** (hydrolysed): exp 27,768.18 Da, act 27,767.80 Da. a.u., arbitrary units. Source data

Next, we explored reassigning codons to hydroxy acids with a view towards the encoded cellular synthesis of depsipeptide macrocycles. The *Mm* and *M. barkeri* (*Mb*) pyrrolysyl-tRNA synthetases and their cognate tRNAs have been used to incorporate four lysine hydroxy acid derivatives—with limited efficiency at single sites in proteins^19–21^. We set out to (1) reassign the TCG sense codon in Syn61Δ3(ev5) to hydroxy acids and to (2) increase the chemical diversity of distinct hydroxy acids (Fig. 2a) that can be incorporated—via cellular translation—into protein and peptide sequences.

Using a liquid chromatography mass spectrometry (LC–MS) assay, we demonstrated that BocK-OH (**6**), a representative aliphatic hydroxy acid, is taken up by *E. coli* when added to the growth media (Fig. 2b); we observed a substantial conversion of BocK-OH into the corresponding amino acid. We expressed sfGFP-3-TCG (a superfolder green fluoroescence protein (sfGFP) gene that contains a TCG codon at position 3 and no other TCG codons within the gene) in Syn61Δ3(ev5) cells that contained *Mm*PylRS/*Mm*tRNA^Pyl^_CGA_ supplemented with 2 mM BocK-OH; ESI–MS, under buffer conditions that induce ester bond cleavage, demonstrated selective BocK-OH incorporation (Fig. 2c). Thus, despite substantial intracellular conversion of BocK-OH into the corresponding amino acid, we observed the selective incorporation of BocK-OH with the *Mm*PylRS/*Mm*tRNA^Pyl^_CGA_ pair. Notably, we observed very little, if any, intracellular conversion of BocK into BocK-OH (Fig. 2b). We conclude that when BocK-OH is added to cells there is a substantial metabolic conversion into BocK, and the *Mm*PylRS/*Mm*tRNA^Pyl^ pair selectively incorporates BocK-OH into proteins in preference to BocK. However, when BocK is added to cells it is not substantively converted into BocK-OH and the *Mm*PylRS/*Mm*tRNA^Pyl^ pair incorporates BocK. Both these results are consistent with prior observations, which demonstrated that the *Mm*PylRS/*Mm*tRNA^Pyl^_CUA_ pair can incorporate amino acids or hydroxy acids when these compounds are provided as substrates^19,20^. However, our results demonstrate that these prior observations are the result of: (1) asymmetry in the cell’s metabolism—the hydroxy acid is converted into the amino acid, but the amino acid is not substantively converted into the hydroxy acid—and (2) the selectivity of *Mm*PylRS for the hydroxy acid.

Next, we synthesized seven additional hydroxy acids (**7**–**13**) with aliphatic side chains (Fig. 2a); **6**, **7** and **12** were previously incorporated with PylRS variants^19,21,22^. We investigated the incorporation of each hydroxy acid (**6**–**13**) with the *Mm*PylRS/*Mm*tRNA^Pyl^_CGA_ pair, the *1R26*PylRS/*Alv*tRNA^ΔNPyl(8)^_CUA_ pair^18^ and several active-site variants of each pair. We identified aaRS/tRNA pairs that led to robust—hydroxy acid dependent—increases in sfGFP-3-TCG or sfGFP-3-TAG expression (Fig. 2d). To confirm the selective incorporation of each hydroxy acid into sfGFP with a cognate aaRS/tRNA pair, we purified the protein and measured its mass by ESI–MS under buffer conditions that induced ester bond cleavage. We observed selective hydroxy acid incorporation in all cases (Fig. 2e). Notably, when cells that contained PylRS/tRNA_CUA_ pairs and the sfGFP-3-TAG were supplemented with both 2 mM amino acid and 2 mM hydroxy acid (for **6**, **7** and **12** and their respective amino acid analogues), we observed only hydroxy acid incorporation into sfGFP (Supplementary Fig. 3). These results are consistent with PylRS systems being selective for hydroxy acids, with aliphatic side chains, over the corresponding amino acids.

### Genetically encoding hydroxy acids with aromatic side chains

**Fig. 3:**
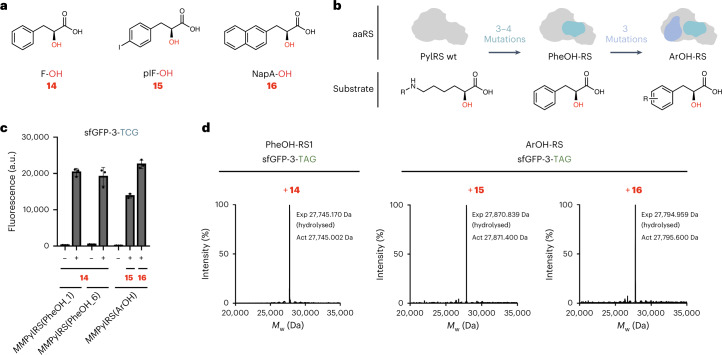
Directed evolution of PylRS variants with specificity for hydroxy acids with aromatic side chains. **a**, Structures of hydroxy acids with aromatic side chains attached to the beta carbon, **14**–**16**, used in this study. **b**, Strategy to generate PylRS variants with specificity towards hydroxy acids with aromatic side chains. Directed evolution of *Mm*PylRS (light grey) yielded variants *Mm*PylRS(PheOH_1) and *Mm*PylRS(PheOH_6), which selectively incorporate **14** (phenyllactic acid). Further directed evolution of *Mm*PylRS(PheOH_6), using libraries that mutate amino acids in the enzyme involved in recognizing the side chain of the substrate led to the discovery of ArOH-RS. This variant enables the incorporation of hydroxy acids **15** and **16** that bear substituents on the phenyl ring. **c**, Activity of *Mm*PylRS(PheOH_1), *Mm*PylRS(PheOH_6) and *Mm*PylRS(ArOH) with hydroxy acids that bear aromatic side chains (**14**–**16**). The production of sfGFP was measured in Syn61∆3(ev5) cells that contained the indicated aaRS and cognate tRNA, an sfGFP-3-TCG gene and the indicated hydroxy acid. Error bars represent standard deviation from the mean of three biological replicates. **d**, sfGFP-His6 was produced in the presence of **14**, **15** and **16** in DH10B cells transformed with sfGFP-3-TAG, the indicated aaRS and cognate tRNA. The identity of the monomer incorporated was verified by ESI–MS. In all three cases, only the mass that corresponded to hydroxy acid incorporation followed by hydrolysis of the ester bond at position 3 was detected, which confirmed the selectivity of each aaRS for the indicated hydroxy acid. Source data

Next, we aimed to incorporate hydroxy acids with aromatic side chains (Fig. 3a). We tested the *Af*TyrRS(pIF)/*Af*tRNA^Tyr(A01)^_CUA_ pair for its ability to incorporate the corresponding hydroxy analogue of pIF, (*S*)-2-hydroxy-3-(4-iodophenyl)propanoic acid (pIF-OH) (**15**), and the *Methanocaldococcus jannaschii* (*Mj*)TyrRS(Nap)/*Mj*tRNA^Tyr^_CUA_ pair, which can incorporate l-3-(2-naphthyl)alanine)^23^, for its ability to incorporate the corresponding hydroxy analogue, (*S*)-2-hydroxy-3-(naphthalen-2-yl)propanoic acid (NapA-OH) (**16**). ESI–MS analysis of sfGFP purified from cells that contained each aaRS/tRNA pair, the corresponding hydroxy acid and sfGFP-3-TAG revealed incorporation of the corresponding amino acid into sfGFP (Supplementary Fig. 4). We demonstrated that these aromatic hydroxy acids are converted into the corresponding amino acids in cells (Supplementary Fig. 4). Previous work reported that deletion of two transaminases *aspC* and *tyrB* in *E. coli* enables the biosynthetic accumulation of *p-*hydroxy-l-phenyllactic acid at sufficient levels to direct its incorporation using an engineered *Mj*TyrRS/*Mj*tRNA^Tyr^_CUA_ pair^24^. Disrupting these two transaminases slightly increased the intracellular levels of added **15** and **16**, but did not stop their conversion into the corresponding amino acids. Moreover, sfGFP purified from *ΔaspC/ΔtyrB* double-knockout cells, which contained the *Af*TyrRS(pIF)/*Af*tRNA^Tyr(A01)^_CUA_ or the *Mj*TyrRS(Nap)/*Mj*tRNA^Tyr^_CUA_ pair, **15** or **16**, and sfGFP-3-TAG, exclusively incorporated the corresponding amino acid analogues (Supplementary Fig. 4).

We hypothesized that TyrRS derivatives are selective for amino acids with aromatic side chains over the corresponding hydroxy acids; this hypothesis is consistent with structures of *Af*TyrRS in complex with tyrosine, which show that the amino group of tyrosine is specifically coordinated by Y158, Q162 and Q180 residues in the enzyme^25^. Because the *Mm*PylRS/*Mm*tRNA^Pyl^ pair is selective for aliphatic hydroxy acids over the corresponding amino acids, and aromatic amino acids have been incorporated using engineered mutants of this pair^26^, we hypothesized that it might be possible to engineer the *Mm*PylRS/*Mm*tRNA^Pyl^ pair for the cellular incorporation of hydroxy acids with aromatic side chains, despite the metabolic interconversion of the hydroxy acids into amino acids (Fig. 3b). To achieve this, we constructed a *Mm*PylRS library by randomizing the codons at five positions in the active site (M300, L301, A302, M344 and N346). These residues are proximal to the substrate’s α-amino group, and are involved in preventing the binding of phenylalanine to *Mm*PylRS^27^. We subjected the library to one round of positive selection in the presence of **14**, the hydroxy acid analogue of phenylalanine; the selection was based on the ability to read through an amber stop codon in a chloramphenicol acetyltransferase gene (CAT-D111-TAG) and confer resistance to chloramphenicol^28^. We screened the resulting *Mm*PylRS/*Mm*tRNA^Pyl^_CUA_ derivatives to identify clones that generated robust GFP fluorescence from sfGFP-3-TAG in the presence of **14**, but generated minimal fluorescence in the absence of **14**; this yielded six mutants, *Mm*PylRS(PheOH_1) to *Mm*PylRS(PheOH_6), which directed the selective incorporation of **14** (Fig. 3c and Supplementary Fig. 5).

**Fig. 4:**
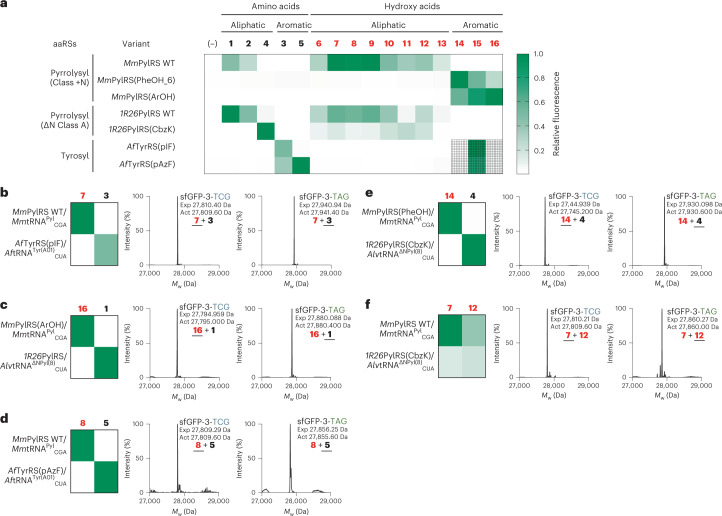
Defining the ncAA and hydroxy acid specificity of aaRSs derived from mutually orthogonal pairs. **a**, Seven orthogonal aaRS variants and their cognate tRNAs, which span three mutually orthogonal aaRS classes, were tested for their ability to incorporate 16 monomers (5 ncAAs and 11 alpha hydroxy acids with aliphatic and aromatic side chains) into sfGFP. The fluorescent protein was expressed from sfGFP-3-XXX (where XXX is TCG for Class +N pyrrolysyl-derived pairs, and TAG for ΔN Class A pyrrolysyl-derived pairs and tyrosyl-derived pairs) in Syn61∆3(ev5) cells. Fluorescence levels within a row are normalized to the maximum activity of the active-site variant assayed in that row; raw data are provided in Supplementary Fig. 6b–h. Hashed squares indicate that the data results from amino acid incorporation in cells supplemented with the corresponding hydroxy acid. *1R26*PylRS(CbzK) is *1R26*PylRS-Y126G-M129L^18^, *Af*TyrRS(pIF) is *Af*TyrRS^−^Y36I-L69M-H74L-Q116E-D165T-I166G^17^ and *Af*TyrRS(pAzF) is *Af*TyrRS-Y36T-H74L-Q116E-D165T-I166G-N190K^17^. **b**–**f**, Further characterizing five pairs of mutually orthogonal active sites derived from the different synthetase types (Class + N pyrrolysyl, ΔN Class A pyrrolysyl and tyrosyl) shown in **a**. In the presence of both aaRS/tRNA pairs and their cognate substrates (**b**, *Mm*PylRS WT and *Af*TyrRS(pIF) with **7** and **3**; **c**, *Mm*PylRS(ArOH) and *1R26*PylRS WT with **16** and **1**; **d**, *Mm*PylRS WT and *Af*TyrR(pAzF) with **8** and **5**; **e**, *Mm*PylRS(PheOH) and *1R26*PylRS(CbzK) with **14** and **4**; **f**, *Mm*PylRS WT and *1R26*PylRS(CbzK) with **7** and **12**), selective incorporation of each monomer by each pair was confirmed by ESI–MS of the sfGFP expressed from sfGFP-3-TCG or sfGFP-3-TAG reporters. The underlined monomer was selectively incorporated. WT, wild type. Source data

Next, to encode further alpha hydroxy acids with aromatic side chains (**15** and **16**), we created saturation mutagenesis libraries based on *Mm*PylRS(PheOH_1), *Mm*PylRS(PheOH_3) and *Mm*PylRS(PheOH_6). These libraries targeted three residues (C348, V401 and W417) that delimit the pocket in the enzyme that binds to the side chain of its substrates. We subjected the pooled libraries to positive selection on chloramphenicol, in the presence of **15** or **16**, using the CAT-D111-TAG based selection. We further analysed the surviving clones from the selection in the presence of **15**, using an sfGFP-3-TAG reporter, for their activity in the presence and absence of **15** and **16**. We thereby identified one variant*, Mm*PylRS(ArOH), derived from *Mm*PylRS(PheOH_6), with the desired properties. The *Mm*PylRS(ArOH)/*Mm*tRNA^Pyl^_CUA_ pair selectively incorporated **15** or **16** to produce sfGFP from sfGFP-3-TAG in *E. coli* cells and exhibited little activity towards phenylalanine or the amino acid analogues of **15** and **16** (Figs. 3c,d and 4a, and Supplementary Fig. 5). We demonstrated that the active site of ArOH-RS (as well as of PheOH-RS1 and PheOH-RS6) effectively discriminated against hydroxy acids with aliphatic side chains (compounds **6**–**13**) (Fig. 4a).

### Mutually orthogonal pairs to encode ncAAs and hydroxy acids

Class +N pyrrolysyl pairs (which include *Mm*PylRS/*Mm*tRNA^Pyl^), ΔN Class A pyrrolysyl pairs (which include *1R26*PylRS/*Alv*tRNA^ΔNPyl(8)^) and the *Af*TyrRS/*Af*tRNA^Tyr^ pair or *Mj*TyrRS/*Mj*tRNA^Tyr^ pair are mutually orthogonal in their aminoacylation specificity^17,18^. To use combinations of these pairs to encode distinct non-canonical monomers, they must be engineered to specifically decode distinct codons and specifically recognize distinct non-canonical monomers. Pyrrolysyl tRNAs have been engineered to recognize many different codons^29^ and tyrosyl tRNAs directed to the amber codon^30^; therefore, we anticipated that all combinations of distinct pairs could be directed to decode TAG and TCG codons. Thus, the outstanding challenge was to define mutually orthogonal non-canonical monomer specificity for Class +N pyrrolysyl pairs, ΔN Class A pyrrolysyl pairs and the tyrosyl pairs.

We expressed each aaRS/tRNA pair, and several active-site variants, with 5 ncAAs and 11 hydroxy acids, and we measured production of sfGFP from sfGFP-3-XXX (where XXX is TAG for ΔN Class A pyrrolysyl-derived pairs and tyrosyl-derived pairs, and TCG for Class +N pyrrolysyl-derived pairs) in Syn61Δ3(ev5) (Fig. 4a). From the matrix of 116 combinations of aaRS/tRNA pairs and monomers we directly identified 77 pairs of mutually orthogonal active sites (and pairs of cognate monomers) in synthetases that are mutually orthogonal in their tRNA specificity (Fig. 4b–e and Supplementary Fig. 6). We also identified 49 unique reassignment schemes (Supplementary Table 1). We focused on further characterizing a subset of these aaRS/tRNA pair combinations as the basis of additional reassignment schemes for encoding a ncAA and a hydroxy acid or for encoding two hydroxy acids (Fig. 4b–f). Within most of the pairs of aaRS/tRNA pairs that we characterized in detail, each aaRS directs the incorporation of its cognate monomer to synthesize full-length sfGFP, but minimally directs the incorporation of the monomer that is efficiently incorporated by the other aaRS/tRNA pair (Fig. 4b–e). In addition, when cells that contain both aaRS/tRNA pairs were provided with both non-canonical monomers, we observed selective incorporation of the cognate monomer into sfGFP by each aaRS/tRNA pair, as judged by mass spectrometry (Fig. 4b–e). These observations indicated that these Class +N pyrrolysyl pairs, ΔN, Class A pyrrolysyl pairs and tyrosyl pairs have active sites with mutually orthogonal specificity for specific combinations of ncAAs and hydroxy acids, as required to incorporate a ncAA and a hydroxy acid into a single genetically encoded polymer within the cell.

We noted that the *Mm*PylRS/*Mm*tRNA^Pyl^_CGA_ pair efficiently incorporated **1** (AllocK) but not **4** (CbzK), and that the same pair incorporated both **7** (AllocK-OH) and **12** (CbzK-OH). Similarly, we observed that the *1R26*PylRS(CbzK)/*Alv*tRNA^ΔNPyl(8)^_CUA_ pair efficiently incorporated **4** (CbzK) but not **1** (AllocK), and that the same pair incorporated both **12** (CbzK-OH) and **7** (AllocK-OH) (Fig. 4a). We hypothesized that the intrinsic preference of *Mm*PylRS for the AllocK side chain in **1** over the CbzK side chain in **4** might be preserved in the recognition of the corresponding hydroxy acids. Similarly, we hypothesized that the intrinsic preference of *1R26*PylRS(CbzK) for the CbzK side chain in **4** over the AllocK side chain in **1** might be preferred in the recognition of the corresponding hydroxy acids. Indeed, when cells that contain both the *Mm*PylRS/*Mm*tRNA^Pyl^_CGA_ pair and the *1R26*PylRS(CbzK)/*Alv*tRNA^ΔNPyl(8)^_CUA_ pair were provided with both **7** and **12**, we observed selective incorporation of **7** in response to the TCG codon in sfGFP-3-TCG and selective incorporation of **12** in response to the TAG codon in sfGFP-3-TAG (Fig. 4f and Supplementary Fig. 6). These observations indicated that these Class +N pyrrolysyl pairs and ΔN, Class A pyrrolysyl pairs have active sites with mutually orthogonal hydroxy acid specificity, as required to incorporate two distinct hydroxy acids into a single genetically encoded polymer within the cell.

### Combinatorially encoding hydroxy acids in depsipeptides

**Fig. 5:**
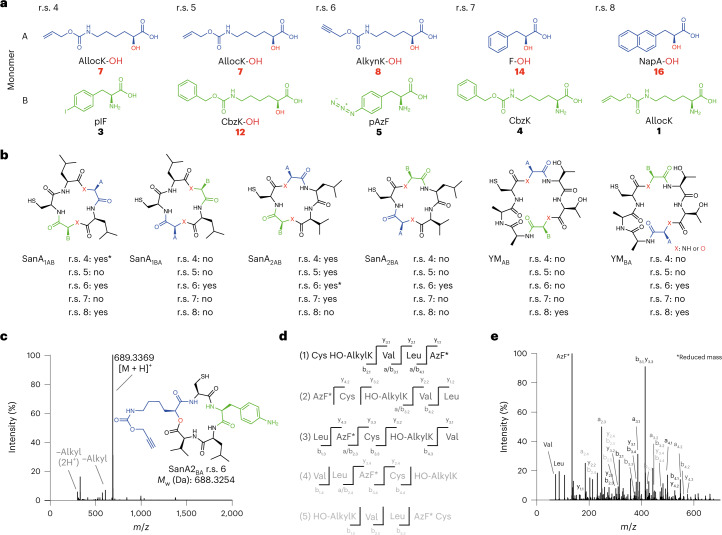
Genetically programmed cell-based synthesis of non-natural depsipeptide macrocycles. **a**, r.s. 4–8 used for macrocyclic depsipeptide synthesis. The reassignment schemes define the identity of monomers A and B; monomer A (blue) is incorporated in response to the TCG codon and monomer B (green) is incorporated in response to the TAG codon. **b**, The chemical structure of all the cyclic depsipeptides indicate the positions of non-canonical monomers A and B. For r.s. 4–8, ‘yes’ indicates that we detected the exact mass of the depsipeptide after its purification, ‘no’ indicates that we could not detect the peptide and the asterisk indicates hydrolysis of the ester bond after successful cyclization. The raw mass spectra are provided in Supplementary Fig. 7. **c**, Mass spectrum and chemical structure of the depsipeptide SanA_2BA_ r.s. 6 with known fragment peaks annotated relative to the [M + H]^+^ peak; trace hydrolysis was observed (Supplementary Fig. 7m). **d**,**e**, Expected linear sequences for the fragmentation of the cyclic peptide SanA_2BA_ r.s. 6 (**d**); all the detected a-, b- and y-series fragments are annotated in the tandem mass spectra (**e**).

To synthesize macrocyclic depsipeptides we combined the macrocycle-encoding gene fusions (Fig. 1) with r.s. 4–8 (Fig. 5); these reassignment schemes were defined based on the aaRS/tRNA specificity we defined (Fig. 4). In r.s. 4, monomer A is **7** (AllocK-OH), incorporated using the *Mm*PylRS/*Mm*tRNA^Pyl^_CGA_ pair, and monomer B is **4** (pIF), incorporated using the *Af*TyrRS(pIF)/*Af*tRNA^Tyr(A01)^_CUA_ pair. In r.s. 5, monomer A is **7** (AllocK-OH), incorporated using the *Mm*PylRS/*Mm*tRNA^Pyl^_CGA_ pair, and monomer B is **12** (CbzK-OH), incorporated using the *1R26*PylRS(CbzK)/*Alv*tRNA^ΔNPyl(8)^_CUA_ pair. In r.s. 6, monomer A is **8** (AlkynK-OH), incorporated using the *Mm*PylRS/*Mm*tRNA^Pyl^_CGA_ pair, and monomer B is **5** (pAzF), incorporated using the *Af*TyrRS(*p*AzF)/*Af*tRNA^Tyr(A01)^_CUA_ pair. In r.s. 7, monomer A is **14** (F-OH), incorporated using the *Mm*PylRS(PheOH_6)/*Mm*tRNA^Pyl^_CGA_ pair, and monomer B is **5** (CbzK), incorporated using the *1R26*PylRS/*Alv*tRNA^ΔNPyl(8)^_CUA_ pair. In r.s. 8, monomer A is **16** (NapA)-OH, incorporated using the *Mm*PylRS(ArOH)/*Mm*tRNA^Pyl^_CGA_ pair, and monomer B is **1** (AllocK), incorporated using the *1R26*PylRS/*Alv*tRNA^ΔNPyl(8)^_CUA_ pair. These new reassignment schemes introduce non-natural side chains and also introduce one or two ester bonds into the backbone of the macrocycle (Fig. 5a,b).

We produced, isolated and confirmed the exact mass for 12 depsipeptides (Fig. 5b and Supplementary Fig. 7) and 10 of these isolated depsipeptides were macrocyclic; note that for 3 of these 10 depsipeptides we observed a fraction of the molecules that were not cyclized (Supplementary Fig. 8). Two depsipeptides were isolated as hydrolysed linear products in which the ester bond was cleaved (Fig. 5a)—this hydrolysis is likely to be sequence dependent. We further analysed the depsipeptide produced from SanA_2BA_ with r.s. 6 (Fig. 5c) by tandem mass spectromety. The cyclic molecule can theoretically fragment into five linear sequences (Fig. 5d), which can further fragment. We identified at least three fragments for each of the five linear sequences for this molecule, which further confirmed that the isolated depsipeptide is cyclic and has the expected sequence of monomers (Fig. 5d,e).

## Discussion

We demonstrated the programmable cell-based synthesis of macrocyclic peptides that contain ncAAs and macrocyclic depsipeptides that contain non-canonical side chains and one or two ester bonds. By changing the gene sequence we programmed non-canonical monomers at different positions in the macrocycles, and by changing the reassignment scheme we changed the identity of the monomers encoded at each position.

In future work, we aim to realize the cell-based, encoded synthesis of an even wider range of non-canonical polymers and macrocycles. To encode an even wider range of non-natural side chain and backbone functionality, we will further expand the substrate scope of orthogonal aaRSs and orthogonal ribosomes^31^. We aim to genetically encode more of the diverse non-canonical structures accessed by non-ribosomal peptide synthetase and RiPPs^3,4^, as well as non-canonical structures that are currently unknown in natural biology. We note that the encoded cell-based synthesis of non-canonical macrocycles may provide a foundation for the directed evolution and selection of non-canonical macrocycles with new functions^32,33^.

## Methods

### Gene recoding

For experiments in Syn61Δ3-derived cells, for all the genes and plasmids it was necessary to compress the genetic code according to the recoding rules of Syn61^12^. For all the protein coding sequences, we recoded TCG to AGC, TCA to AGT and TAG to TAA using a custom Python script^12^. For all the plasmids used in this study, please see Supplementary Table 2 for the sequence details.

### Construction of aaRS/tRNA plasmids to decode TCG or TAG codons in Syn61Δ3

To incorporate a single ncAA, we used a pMB1-based plasmid that encoded the orthogonal aaRS/tRNA pair that directs ncAA incorporation in response to the TCG or TAG codons. For *Mm*tRNA^Pyl^_CGA_ constructs, which incorporate aliphatic monomers in response to the TCG codon, we designed *pylT* tRNA genes, which included *pylT*opt mutations^37^ and which replaced the entire anticodon stem loop (the anticodon plus six nucleotides on either side) with the anticodon stem loop sequence of the *serU* (in the case of CGA) *E. coli* gene^13^.

We constructed pMB1-based aaRS/tRNA plasmids by the HiFi Assembly of multiple fragments, which included (1) a recoded aaRS under the control of the constitutive *glnS* promoter, synthesized as a gBlock (IDT), and (2) a recoded pMB1 plasmid backbone generated via PCR from previous recoded aaRS/tRNA plasmids^13^; this backbone included a recoded *kan*^*R*^ gene, a pMB1 vector origin and an orthogonal tRNA gene with an *lpp* promoter and an *rrnC* terminator. With this assembly approach we generated all the novel aaRS/tRNA plasmids from this study. See Supplementary Table 2 for sequence details.

For double distinct ncAA incorporations, we designed pMB1-based plasmids which polycistronically encoded two aaRS/tRNA pairs^13,38^. Briefly, we added two aaRS coding sequences downstream of a *glnS* promoter, for which we optimized the intergenic region between the aaRS genes with an RBS calculator program (https://salislab.net/software/). For the polycistronic tRNA operon, we placed both tRNA genes downstream of an *lpp* promoter; the intergenic region between tRNA genes was based on the intergenic region between the *alaX* and *alaW* genes in the *E. coli* genome.

### Construction of vectors to express His_6_-SUMO-ncPeptide-GyrA

All the cloning was conducted in *E. coli* DH10B. We generated plasmids to express SUMO-Peptide-GyrA genes based on a recoded pBAD expression system^13^. Macrocycle coding (ncPeptide) sequences were introduced by QuikChange PCR or by the ligation of phosphorylated primers with a PCR-amplified and AraI-digested SUMO-GyrA plasmid backbone. The final plasmid contained (1) a recoded SUMO-ncPeptide-GyrA gene and (2) a recoded pBAD plasmid backbone containing *apra*^*R*^, *araC* and a p15a vector origin region. sfGFP reporter plasmids were generated as described previously^13^. See Supplementary Table 2 for sequence details.

### Generating ΔaspCΔtyrB cells

We used a two-step lambda-red recombineering approach for the scarless deletion of the *aspC* and *tyrB* genes in *E. coli* DH10B cells. Briefly, the first recombination introduced the pheS*-HygR double selection cassette to delete the target gene, and the second recombination introduced a repair template to replace the double selection cassette with the native genomic environment of the targeted locus. We PCR amplified a pheS*-HygR double selection cassette with primers that contained 50 bp flanking homology arms to the genomic landing site of interest. We then integrated the pheS*-HygR cassette at the designated genomic loci in DH10B cells that harboured the pKW20_CDFtet_pAraRedCas9_tracrRNA plasmid expressing the lambda-red alpha/beta/gamma genes. To initiate recombination, we prepared electrocompetent cells and electroporated 3 μg of the purified PCR product into 100 μl of DH10B cells preinduced to express the lambda-red recombination machinery, as described previously^39^. We induced the arabinose promoter-controlled recombination machinery with l-arabinose added at 0.5% for 1 h starting at OD_600_ = 0.2; preinduced cells were then electroporated and recovered for 1 h at 37 °C in 4 ml of super optimal broth medium. Cells were then diluted into 50 ml of LB medium with 10 μg ml^–1^ tetracycline and grown for 4 h at 37 °C, 200 r.p.m. The cells were subsequently spun down, resuspended in 4 ml of H_2_O, serially diluted, plated and incubated overnight at 37 °C on LB agar plates that contained 10 μg ml^–1^ tetracycline and 200 μg ml^–1^ hygromycin. We constructed the *aspC* and *tyrB* repair templates through overlap extension PCR; this was introduced during the second recombination with the same method as described above, except that the LB agar plates contained 2.5 mM *p*-Cl-Phe in place of hygromycin. We verified the scarless deletions of *aspC* and *tyrB* by genotyping with primers that flanked the locus of interest; sequential deletion of *aspC* and *tyrB* through this two-step lambda-red process yielded the DH10B *ΔaspC*/*ΔtyrB* cells. Primers used to generate DH10B *ΔaspC*/*ΔtyrB* cells are provided in Supplementary Table 2.

### Measuring the intracellular levels of amino acids or hydroxy acids

We measured the intracellular concentration of non-canonical amino and hydroxy acids with an LC–MS method previously described^40^. Briefly, we supplemented amino or hydroxy acids (1 mM final concentration) to a 5 ml solution of LB inoculated with diluted DH10B cells; a negative control sample was prepared in parallel without amino or hydroxy acid addition. We grew the samples at 37 °C with shaking (220 r.p.m.) for 12 h. After growth, we measured the OD_600_ of each culture, and we then harvested the cells from each one. We washed, centrifuged and resuspended the cell pellets for three cycles with 1 ml of ice-cold LB media. We then resuspended each washed cell pellet in a methanol–water solution (60:40) and added zirconium beads (0.1 mm) to each suspension. We then lysed the suspensions by vortexing for 12 min, and we obtained the clarified lysate after centrifugation at 21,000*g* for 30 min at 4 °C. We then pipetted the clarified lysate supernatant carefully into a fresh 1.5 ml microcentrifuge tube, and subjected the sample to another round of centrifugation at 21,000*g* for 2 h at 4 °C. For the LC–MS analysis, we transferred a 100 µl aliquot of the supernatant into 250 µl glass inserts (Agilent) and injected 5 µl from the resulting sample into an Agilent 1260 Infinity LC equipped with a Zorbax C18 (4.6 × 150 mm) column. After running the column, 0.5 to 95% acetonitrile in water, the sample was injected into an Agilent 6130 Quadrupole LC–MS unit, with the mass spectrometer set to selected ion monitoring mode to detect the *m*/*z* value for the relevant amino or hydroxy acid.

### sfGFP expression measurements

We expressed sfGFP genes that bear single TCG or TAG codons at position 3 in Syn61Δ3(ev5) cells harbouring plasmids that encoded aaRS/tRNA genes, as described previously^13^. Briefly, we co-electroporated 50 µl of Syn61Δ3(ev5) cells (all expressions utilized Syn61∆3(ev5) cells) with a pMB1-based aaRS/tRNA plasmid (100 ng) and recovered them for 1.5 h in 1 ml of super optimal broth with shaking, 1,000 r.p.m. at 37 °C. Subsequently, we inoculated the cells (1 ml) into 6 ml of 2xYT media supplemented with 50 µg ml^–1^ kanamycin and incubated the cells overnight at 37 °C with shaking at 220 r.p.m. After recovery, we prepared the cells as electrocompetent for the subsequent electroporation of a pBAD_sfGFP reporter plasmid (100 ng) and recovered the cells in deep-well 96-well plates in the presence of 50 µg ml^–1^ kanamycin and 50 µg ml^–1^ apramycin. Similarly, cells were prepared as chemically competent prior to the transformation (described below). After 36 h of recovery, we set-up expressions in a 96-well microtitre plate format, inoculating overnight cultures 1:100 into 0.5 ml of 2xYT that contained kanamycin (50 µg ml^–1^), apramycin (50 µg ml^–1^), l-arabinose (0.2%) and the presence or absence of ncAA. All ncAAs were supplemented to a final concentration of 2 mM, except the AlkynK (**2**) amino acid was supplemented at 7.5 mM, (*S*)-6-acetamido-2-hydroxyhexanoic acid (AcK-OH) (**13**) was supplemented at 10 mM and F-OH (**15**) was supplemented at 4 mM. We incubated the 96-well plates for 16 h at 37 °C with shaking at 750 r.p.m. in a Thermo-Shaker (Grant-bio). We centrifuged the plates for 10 min at 3,200*g* and subsequently resuspended the cell pellets with 150 µl of PBS. To measure the sfGFP expression normalized by cell density, we transferred 100 µl of resuspended cells into a Costar clear 96-well flat-bottom plate, and recorded OD_600_ and GFP fluorescence (*λ*_ex_, 485 nm; *λ*_em_, 520 nm) measurements on a PHERAstar FS plate reader (BMG LABTECH) (gain setting of 0 and focal adjustment of 00 mm).

### Purification of His6x-tagged sfGFP proteins from *E. coli*

*E. coli* cells (Syn61∆3(ev5), DH10B or DH10B *ΔaspC/ΔtyrB*) that harboured a pMB1-based aaRS/tRNA plasmid and a pBAD_sfGFP p15a-based plasmid were grown for 16 h with shaking (220 r.p.m.) at 37 °C in the same media conditions as those described for the sfGFP expression measurements (see above). To prepare overnight cultures, we used 25 ml volumes for sfGFP-3-TCG or sfGFP-3-TAG. After expression, we centrifuged cultures and resuspended them in lysis buffer (1 × PBS, 1 × Bugbuster Protein Extraction Reagent (Novagen), in a volume of 1/20th that of the original culture, supplemented with 50 µg ml^–1^ DNase 1, 20 mM imidazole and 100 µg ml^–1^ lysozyme. We incubated the cell resuspensions at 4 °C for 30 min and then clarified the lysates by centrifugation for 30 min at 4 °C and 16,000*g*. We subsequently transferred the clarified lysate into 1.5 ml microcentrifuge tubes that contained 50 µl of Ni^2+^–NTA slurry (Qiagen) and incubated the mixture for 1 h while tumbling at 4 °C. After incubation, we collected the Ni^2+^–NTA beads by gravity filtration on a fritted column and resuspended them three times in 500 µl of wash buffer (PBS, 40 mM imidazole, pH 8). For polyhistidine-tagged protein elution, we added 100 µl of elution buffer (PBS, 300 mM imidazole, pH 8) to the beads and centrifuged the mixture (1,000*g*, 4 °C, 1 min) to collect the eluate through the fritted column into a fresh 1.5 ml microcentrifuge tube. We repeated this elution three times and stored the purified sfGFP protein at −20 °C prior to downstream analyses.

### Preparation of chemically competent Syn61Δ3(ev5) cells and heat-shock transformation

An aliquot of 100 ml of LB medium was inoculated with 3 ml of a Syn61Δ3(ev5) overnight culture and grown while agitated (200 r.p.m., 37 °C) to an OD_600_ of 0.3–0.4 (~3 h). The cells were chilled on ice for 10 min and harvested by centrifugation (3,200*g*, 5 min, 4 °C), and the pellet was resuspended in 0.1 M CaCl_2_ and incubated on ice for at least 30 min. The cells were collected as before, resuspended in a total volume of 4 ml of 0.1 M CaCl_2_ supplemented with 10% glycerol and incubated on ice for another 5 min. The cells were aliquoted in 100 µl portions on ice and flash frozen until further use. For heat-shock transformation, the frozen cells were thawed on ice and 40 µl of the cells were mixed with about 100 ng of plasmid DNA. The cells were heat shocked in a water bath (42 °C) for 45 s and then chilled for 5 min on ice. SOC medium (500 µl) was added and the cells were recovered for 1.5 h at 37 °C with shaking at 1,000 r.p.m. The culture was then diluted into 3 ml of selective LB medium with the relevant antibiotic and grown for 36 h with shaking (200 r.p.m.) at 37 °C.

### Site-saturation mutagenesis of aaRS libraries

Site-saturation mutagenesis was performed on the *Mm*PylRS gene in a pMB1 plasmid backbone via enzymatic inverse PCR^41^, using primers that contained a BsaI restriction site (see Supplementary Table 2 for primer details). After restriction digest and religation, libraries were transformed into electrocompetent DH10B cells to ensure a transformation of efficiency of at least 10^9^ clones. Quality control to ensure the library diversity was performed through Sanger sequencing of individual clones.

### aaRS selections and screening

Libraries were transformed into freshly prepared electrocompetent DH10B cells that contained a CAT-D111-TAG–sfGFP-N150-TAG p15A dual selection plasmid to ensure a minimal transformation efficiency of 10^9^. Overnight cultures were diluted in 2xYT media that contained the corresponding hydroxy acid. After growing to OD_600_ = 1.0, cells were plated on varying concentrations of chloramphenicol (which ranged from 100 to 400 µg ml^–1^) in the presence of the corresponding hydroxy acid. Surviving colonies were picked and diluted into media that contained 0.2% l-arabinose in the presence and absence of hydroxy acid, and sfGFP production was monitored as previously stated. Samples that showed hydroxy-acid-dependent sfGFP expression were pooled, and their plasmid DNA was purified. After selectively digesting the CAT-D111-TAG–sfGFP-N150-TAG p15A dual selection plasmid, the aaRS library was retransformed into DH10B cells that harboured sfGFP-3-TAG and plated on 0.2% l-arabinose in the presence of hydroxy acid. Green fluorescent clones were picked, and the individual plasmid DNA was miniprepped (Qiagen). Selectivity and activity for each clone with the corresponding hydroxy acid was monitored by the fluorescence signal after the retransformation with sfGFP-3-TAG.

### Expression of the target peptide as a His6-SUMO-Peptide-GyrA-CBD fusion protein

Chemically competent cells of Syn61Δ3(ev5) were transformed in two steps: (1) the pMB1-based aaRS/tRNA plasmid (kanamycin, 50 µg ml^–1^) was transformed and new chemically competent cells were prepared for each aaRS/tRNA used and (2) these cells were then transformed with each pBAD-based expression plasmid (apramycin, 50 µg ml^–1^). After overnight recovery of the doubly transformed cells in 1.5 ml of super optimal broth, we added 50 ml of selective TB medium. Cells were grown to OD_600_ = 2.5–3.0 (12 h) at 37 °C, 200 r.p.m. We induced expression by the addition of 0.4% l-arabinose and 2 mM each of the ncAA or hydroxy acid (8 mM for F-OH (**15**)), and harvested cells by centrifugation (10 min, 3,200*g*) after another 12 h. We washed the pellets with PBS and stored them at −20 °C. See Supplementary Table 2 for the plasmid details.

### His_6_-SUMO-Peptide-GyrA-CBD purification, cyclization and extraction of macrocyclic (depsi)peptides and LC-MS analysis

Cell pellets were thawed and resuspended in 30 ml of MOPS lysis buffer (20 mM MOPS pH 6.9, 150 mM NaCl, 0.01 mg ml^–1^ DNAse and 0.1 mg ml^–1^ Lysozyme). The resuspended pellet was lysed by sonication in an ice bath (30 × 5 s pulses every 5 s, 50% amplitude) and the cell lysate was cleared from debris by centrifugation (20,000*g*, 25 min, 4 °C). To the supernatant we added 100 µl of Ni^2+^–NTA slurry (50% beads in 20% ethanol) and the suspension was incubated for 1 h at 4 °C under gentle agitation. We collected the Ni^2+^–NTA beads by gravity filtration through a fritted Poly-Prep column (BioRad) and washed with 20 ml of MOPS wash buffer (20 mM MOPS pH 6.9, 300 mM NaCl, 40 mM imidazole). We eluted the protein in 100 µl of MOPS buffer (20 mM MOPS pH 6.9, 150 mM NaCl) supplemented with 200 mM imidazole. We determined the protein concentration after exchanging the buffer for 20 mM MOPS pH 6.9, 150 mM NaCl, by measuring absorption at 280 nm and corrected for the predicted extinction coefficient (ProtParam, Expasy). Before and after the cyclization reaction we mixed 2% of each sample with NuPAGE LDS Sample Buffer (20 µl total, final concentration ×1) and applied a total of 1% elution (10 µl) per lane of a 4–12% Bis-Tris SDS−Gel (NuPAGE); the gel was run at 220 V for 28 min. We stained the gel with InstantBlue (Expedeon) and imaged after destaining with water using the standard setting for Coomassie blue on a ChemiDoc (BioRad).

### Sample preparation for LC–MS analyses of Ulp1 reaction products

For cyclization, we diluted all the purified His_6_-SUMO-Peptide-GyrA-CBD from a single reaction into 500 µL with a MOPS buffer (20 mM MOPS pH 6.9, 150 mM NaCl) and initiated the cyclization reaction by the addition of 0.005 mg ml^–1^ Ulp1 and 100 mM DTT. The reaction was incubated at 37 °C for 18 h. We extracted the resulting cyclic peptides or depsipeptides through the addition of 10% acetic acid (10 µl) and 200 µl of 3:1 chloroform:isopropanol per 500 µl reaction. The sample was mixed thoroughly and the emulsion mixture separated by centrifugation (1 min, 16,000*g*). The bottom organic phase was carefully transferred to a fresh 2 ml Eppendorf tube, and we evaporated the solvent under a strong airflow (within 1 h). The dried film was stored at −20 °C or directly resuspended in 20 µl of 0.2% acetic acid to prepare for LC–MS analyses.

### Fluorescence labelling and quantification of peptide reaction products (YM_AB_ r.s. 1, SanA_1AB_ r.s. 1 and SanA_1AB_ r.s. 2)

The peptides YM_AB_ r.s. 1, SanA_1AB_ r.s. 1 and SanA_1AB_ r.s. 2 were expressed on the 500 ml scale and purified as described above. We exchanged the elution buffer for 20 mM MOPS pH 6.9, 150 mM NaCl, and to determine the protein concentration we measured the absorption at 280 nm and corrected for the predicted extinction coefficient (ProtParam, Expasy). We prepared a control reaction from commercial V5 peptide (Sigma), where 22.5 µg was diluted in a 1 ml reaction buffer (20 mM MOPS pH 6.9, 150 mM NaCl). The Ulp1/2-mercaptoethanesulfonic acid sodium salt (MESNA) excision reaction was initiated by the addition of 50 µl of MESNA (200 mM stock) and 5 µl of Ulp1 (1 mg ml^–1^ stock) to all the samples, and the samples were incubated for 18 h at 37 °C. We collected 20 µl (2%) of the total reaction before and after the incubation with Ulp1/MESNA and mixed with NuPAG LDS Sample Buffer (30 µl total, final concentration ×1). We applied a total of 1% of the SDS sample (15 µl) per lane to a 4–12% Bis-Tris SDS-Gel (NuPAGE), and ran the gel at a current of 220 V for 28 min. The gel was stained with InstantBlue (Expedeon) and imaged after destaining with water using the standard settings for Coomassie blue on a ChemiDoc imager (BioRad). We split the Ulp1/MESNA reaction into two 500 µl samples which were extracted separately with *n*-butanol^42^ or chloroform/isopropanol (3:1). Each sample was acidified with 25 µl of 10% acetic acid and extracted three times with 200 µl of either organic solvent. The organic phase of each extraction was collected and washed once with 5 M NaCl. The water phase was discarded and the organic phase evaporated under vacuum using a Concentrator Plus (Eppendorf *n*-butanol, 45 °C and setting for water 3–4 h; chloroform/isopropanol, room temperature (r.t.) and a highly volatile setting, 15–30 min). We resuspended the dried film in 20 µl of labelling buffer (50 mM Tris pH 8, 5 mM EDTA) by vortexing followed by incubation at 37 °C for 30 min with vigorous shaking (1,000 r.p.m.). The sample was collected by centrifugation (5 min, 16,000*g*) and evenly divided into two amber Eppendorf tubes. To each tube, we added 1 µl of a cysteine reactive fluorophore (100 mM monobromobimane (mBBr), MedChemExpress, or 10 mM Alexa Fluo 488 C5 maleimide, Cy2 similar, Invitrogen); the labelling reaction was left for 1 h at r.t. We quenched the Cy2 reaction for 1 h at r.t. by the addition of 10 mM MESNA; mBBr does not require quenching and was kept at r.t. for another hour. An aliquot of 5 µl of NuPAG LDS Sample Buffer (16 µl total, final concentration ×1.3) was added to each sample and the full samples were separated on a Nove 16% Tricine SDS−Gel (Invitrogen) in parallel with the extracted peptide. A standard curve of labelled V5 peptide was prepared (0–5 nmol, 1:2 dilution) and loaded alongside the sample. A voltage of 120 V was applied for 85 min, then the gel was briefly rinsed with water and immediately imaged. We imaged the Cy2 fluorescence using the standard settings for Cy2 on a Typhoon imager (Amersham), whereas mBBr was imaged using the standard settings for ethidium bromide on a ChemiDoc imager (Bio-Rad). Note that the gels were not stored submerged in water and instead kept wet in a small amount of water. The gel bands were analysed using Fiji (Image J, version 2.1.0/1.53c) built-in gel tools.

### Peptide quantification by gel fluorescence intensity

To quantify peptide abundance, we labelled peptides with the fluorophores mBBr (MedChemExpress) or Alexa Fluor 488 C5 Maleimide (Cy2, Invitroge). Standard curves for Cy2 and mBBr fluorescently labelled peptides were prepared from labelled V5 peptide. We prepared a 1 mM V5 stock solution (Sigma, 1 nmol µl^–1^) in 20 mM Tris, 10 mM EDTA, and used it as an internal standard in all the experiments. We generated in triplicate the standard curves for mBBR- and Cy2-labelled peptides that ranged from 0 to 5 nmol and from 0 to 0.25 nmol, respectively. In amber reaction tubes, we prepared a 30 µl reaction in a ×1 labelling buffer (50 mM Tris pH 8, 5 mM EDTA, ×10 stock) that contained either (1) 0–15 nmol mBBr-labelled V5 peptide from a 10 mM mBBr stock in 100 mM acetonitrile or (2) 0–0.75 nmol Cy2-labelled V5 peptide from a 1 mM Cy2 stock in 10 mM dimethylsulfoxide. After running the reaction for 1 h, we quenched the Cy2 reaction by the addition of 10 mM MESNA (200 mM stock) and incubated both reactions for another hour. We then added ×4 NuPAGE LDS Sample Buffer (×1 final) to all the samples and loaded 13.3 µl of each sample in a Novex 16% Tricine SDS-Gel (Invitrogen) run at 120 V for 85 min. We rinsed the gels briefly with water before imaging. To image Cy2 fluorescence, we used a Typhoon imager (Amersham) set to the standard settings for Cy2, whereas for mBBr imaging we used a ChemiDoc imager (Bio-Rad) set to the standard settings for ethidium bromide. We analysed the bands using the built-in gel tool in Fiji (Image J, version 2.1.0/1.53c). Each individual lane was defined using the built-in tool and the intensity profile of each lane plotted. We used the line tool to define the background and band borders, and we used the wand tool to integrate all the continuous intensity of the target band above the baseline. The standard curve was derived from a linear fit (Prism7, Graphpad) of the measured average band intensity (three replicates) measured for each concentration (0–5 nmol). We used the derived formula to determine the concentration of the detected peptides; the calculated values were corrected for variation using an internal V5 standard with known concentration. A calculated concentration was only given if the Cy2 control showed a visible signal, otherwise a value of 0 µg l^–1^ was given.

### Intact protein and cyclic peptide mass spectrometry

ESI–MS analysis of the proteins and macrocyclic (depsi)peptides was performed using a Waters Xevo G2 mass spectrometer equipped with a modified nanoAcquity LC system. The samples were injected and separated on a BEH C4 UPLC column (1.7 µm, 1.0 × 100 mm, Waters) with a flow rate of 50 µl min^–1^ and a water/acetonitrile gradient from 2% vol/vol to 80% vol/vol (0.1% vol/vol formic acid) over 20 min or, alternatively, desalted and injected directly manually. The eluted sample was directly interfaced via a Zspray electrospray ionization source with a hybrid quadrupole time-of-flight mass spectrometer (Waters). Using a cone voltage of 30 V, data were acquired in the positive ion mode with a range from 300 to 2,000 *m*/*z*. The raw spectra are shown for peptides and protein spectra were deconvoluted using the MaxEnt1 function within MassLynx software (Waters)^43,44^. We used GPMAW (Lighthouse Data) software^45^ to calculate the theoretical WT protein molecular mass and edited them manually to accommodate the molecular mass of ncAAs.

For fragmentation of SanA_2BA_ r.s. 6, the same experimental set-up was used. A gradient of water/acetonitrile (A/B) was used to elute the peptides (1% B for 10 min, to 50% B in 10 min, to 95% B in 2 min, 1 min 95% B, 1 min 99% B, to 1% B, 6 min, flowrate of 50 µl min^–1^), directly interfaced via a Zspray electrospray ionization source with a hybrid quadrupole time-of-flight mass spectrometer (Waters). The target ion (689.3 Da) was selected and fragmented (collision energy, 6 eV), tandem mass spectra were collected over a *m*/*z* range of 50–2.000 *m*/*z*. The raw fragmentation spectrum was compared and assigned manually to the theoretical a, b and y series ions of the five possible linear sequences predicted by MS-Product (http://prospector.ucsf.edu).

### Automated identification of orthogonal aaRS active-site pairs against a panel of non-canonical monomers

For the automated detection of orthogonal pairs from raw data a computational script was employed. Briefly, for aaRS1–monomer 1 to be considered a pair with aaRS2–monomer 2, aaRS1 must be active with monomer 1 and inactive with monomer 2, whereas aaRS2 must be active with monomer 2 but inactive with monomer 1. After obtaining single-codon sfGFP-3-TCG or sfGFP-3-TAG expression data in the presence of amino acids or hydroxy acids, the thresholds used for activity and inactivity were above 10,000 RFU/OD_600_ and below RFU/OD_600_, respectively. aaRSs were grouped in the following way, so that pairs cannot be found with both aaRSs in the same group. Group 1 (pyrrolysyl + N class) comprised *Mm*PylRS, *Mm*PylRS(PheOH_6), *Mm*PylRS(ArOH), group 2 (pyrrolysyl ΔN class A) comprised *1R26*PylRS, *1R26*PylRS(CbzK) and group 3 (tyrosyl class) comprised *Af*TyrRS(pIF) and *Af*TyrRS(pAzF). Data for F-OH, pIF-OH and NapA-OH with *Af*TyrRS(pIF) and *Af*TyrRS(pAzF) were excluded from the analysis, as we demonstrated that these hydroxy acid substrates in the presence of these tyrosyl aaRSs led only to the incorporation of the amino acid and not of the hydroxy acid. We wrote a custom script in Python that takes a matrix of raw data values and identifies tRNA-synthetase/substrate pairs. The script iterates over raw data points for each aaRS–substrate experiment and searches for another aaRS–substrate that abides by the activity and inactivity thresholds defined above; this script is available at https://github.com/JWChin-Lab. Using these criteria, 77 aaRS–substrate pairs were identified, with 49 unique substrate pairings (Supplementary Fig. 8 and Supplementary Table 1).

### Chemical syntheses: general methods

All chemicals and solvents were purchased from Merck, Alfa Aesar or Fisher Scientific and used without further purification. Qualitative analysis by thin layer chromatography was performed on aluminium sheets coated with silica (Merck TLC 60F-254). The spots were visualized under a short-wavelength ultraviolet lamp (254 nm) or stained with basic aqueous potassium permanganate, ethanolic ninhydrin or vanillin. Flash column chromatography was performed with the specified solvent systems on silica gel 60 (mesh 230–400). Medium-pressure LC was performed on a Grace Reveleris X2, equipped with a C18, 120 g, 40 μm prepacked column.

LC–MS analysis was performed on an Agilent 1260 machine. The solvents used consisted of 0.2% formic acid in water (buffer A) and 0.2% formic acid in acetonitrile (buffer B). LC was performed using an Agilent EC-C18 Poroshell 120 (50 × 3 mm, 2.7 μm) and monitored using variable wavelengths.

Mass spectrometry analysis after the LC was carried out in multimode ESI and atmospheric pressure chemical ionization on a 6130 quadrupole spectrometer and recorded in both the positive and negative ion modes. NMR analysis was carried out on a Bruker 400 MHz instrument. All the reported chemical shifts (*δ*) relative to trimethylsilane were referenced to the residual protons in the deuterated solvents used: *d*_1_, chloroform (^1^H *δ* = 7.26 ppm, ^13^C *δ* = 77.16 ppm), *d*_6_, dimethylsulfoxide (^1^H *δ* = 2.49 ppm, ^13^C *δ* = 39.52 ppm), D_2_O (^1^H *δ* = 4.70). Attached proton tests or two-dimensional experiments (correlated spectroscopy and heteronuclear single quantum correlation) were always performed to provide additional information used for the analysis where needed. Coupling constants are given in Hz and described as s (singlet), d (doublet), t (triplet), q (quartet), br (broad singlet), m (multiplet), dd (doublet of doublets) and so on, and combinations thereof.

### Synthesis of hydroxy acids

The hydroxy acids BocK-OH, AllocK-OH and CbzK-OH were all synthesized in accordance with published literature protocols^46^.

#### (*S*)-6-Amino-2-hydroxyhexanoic acid

**Extended Data Fig. 1:**
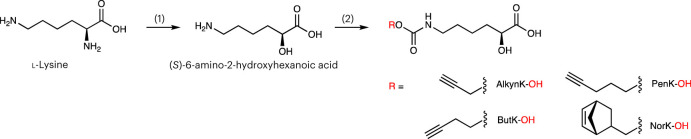
General synthesis of aliphatic hydroxy acids. Reagents and conditions: **1**. NaNO_2_, 10% H_2_SO_4_ (aq), 50 °C, 41% yield; **2**. Lys-OH, corresponding chloroformate, 1M NaOH, THF, 0 °C to RT.

A solution of l-lysine (10 g, 54.8 mmol, 1 equiv.) in 10% H_2_SO_4_ (110 ml) was heated to 50 °C and stirred. NaNO_2_ (12.8 g, 186.2 mmol, 3.4 equiv.) was dissolved in H_2_O (40 ml), and then added to the lysine solution dropwise over 1 h. The reaction was stirred at 50 °C for 3 h. The reaction was quenched by the addition of urea (21 g in 60 ml of H_2_O). The resultant solution was neutralized to pH 7 with the addition of 1 M NaOH, and then acidified to pH 3 with formic acid. H_2_O and excess formic acid were removed under a reduced pressure to concentrate the crude mixture to approximately 50 ml. Amberlite IR120 hydrogen form strongly acidic resin (100 g, Fluka) was charged to a glass column and first washed with H_2_O to a constant pH of ~7, then with 1 M NH_4_OH (200 ml), then with H_2_O to a constant pH of ~7, then with 1 M HCl (200 ml) and finally with H_2_O to a constant pH of ~5. The crude hydroxy acid solution (~50 ml) was loaded to the column and allowed to equilibrate at r.t. for 5 min. The flow through was collected and the resin washed with H_2_O to a constant pH of 5. The product was then eluted with 1 M NH_4_OH (200 ml) and concentrated to give a yellow oil. This oil was dissolved in hot MeOH and allowed to cool to r.t., which resulted in a precipitate that was collected by filtration and dried under reduced pressure. This gave the desired product (*S*)-6-amino-2-hydroxyhexanoic acid as a colourless solid (3.3 g, 41% yield). ^1^H NMR analysis *δ*_H_ (400 MHz, D_2_O) 3.98 (dd, *J* = 6.8, 4.5 Hz, 1H), 2.94 (t, *J* = 7.5 Hz, 2H), 1.83–1.52 (m, 4H), 1.50–1.19 (m, 2H). Low-resolution mass spectroscopy (LRMS) *m*/*z* (ES^+^) 148 [M + H]^+^. See Extended Data Fig. 1.

#### General protocol for the synthesis of AlynK-OH, ButK-OH, PenK-OH and NorK-OH

Typically (*S*)-6-amino-2-hydroxyhexanoic acid (250 mg, 1.7 mmol, 1.2 equiv.) was dissolved in 1 M NaOH(aq.) (2 ml) and THF (1 ml) and the solution stirred and cooled to 0 °C. The corresponding chloroformate (1 equiv.) was added dropwise over 5 min and the reaction allowed to warm to r.t. and stirred for an additional hour. After this time the reaction was diluted with 1 M HCl (20 ml) and extracted with ethyl acetate (3 × 20 ml). The combined organic layers were dried over Na_2_SO_4_, filtered and concentrated. The crude hydroxy acids were typically purified by silica gel column chromatography eluting with ethyl acetate, hexane and acetic acid (50:48:2). This gave pure hydroxy acids as colourless oils. AlynK-OH (285 mg, 89 % yield), LRMS *m*/*z* (ES^–^) 228 [M − H]^–^; ButK-OH (307 mg, 90% yield), LRMS *m*/*z* (ES^–^) 242 [M − H]^–^; PenK-OH (325 mg, 90 % yield), LRMS *m/z* (ES^-^) 256 [M − H]^-^ and NorK-OH (367 mg, 88% yield) LRMS *m/z* (ES^-^) 296 [M − H]^-^.

For ButK-OH, PenK-OH and NorK-OH the chloroformates were premade by the addition of the corresponding alcohols (1.4 mmol, 1 equiv.; 3-butyn-1-ol (Merck 130850), 4-pentyn-1-ol (Merck 302481) and 5-norbornene-2-methanol (Merck 248533) mixture of *endo* and *exo*) in THF (1.5 ml) to a solution of phosgene (737 μl, 1.4 mmol, 1 equiv., 20% in toluene) at 0 °C via a syringe pump over 1 h. The resulting solution was used directly without further purification. See Extended Data Fig. 1.

#### Ack-OH

**Extended Data Fig. 2:**
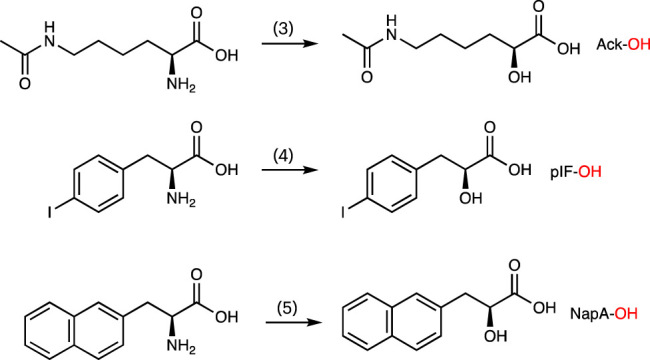
Synthesis of aliphatic hydroxy acid AcK-OH and aromatic hydroxy acids pIF-OH and NapA-OH. Reagents and conditions: **3**. AcK, NaNO_2_, AcOH, H_2_O, RT, 52% yield; **4**. pIF, NaNO_2_, AcOH, H_2_O, RT, 47% yield; **5**. NapA, NaNO_2_, AcOH, H_2_O, RT, 47% yield.

A solution of *N*ε-acetyl-l-lysine (5.0 g, 26.6 mmol, 1 equiv.) in AcOH (10 ml) and H_2_O (40 ml) was stirred at r.t. NaNO_2_ (9.2 g, 132.8 mmol, 5 equiv.) in H_2_O (25 ml) was then added to the amino acid solution dropwise over 30 min. The reaction was stirred at r.t. overnight. Then 1 M HCl (50 ml) was added and the mixture extracted with ethyl acetate (3 × 50 ml). The combined organic layers were dried over Na_2_SO_4_, filtered and concentrated. The crude hydroxy acid was purified by C18 reverse phase medium-pressure LC, eluting with H_2_O/acetonitrile (95/5 to 5/95 over 60 min). The combined product fractions were concentrated to dryness to give pure AcK-OH (2.6 g, 52% yield) as a colourless oil. ^1^H NMR analysis *δ*_H_ (400 MHz, D_2_O) 4.22 (dd, *J* = 7.6, 4.5 Hz, 1H), 3.11 (t, *J* = 6.7 Hz, 2H), 1.90 (s, 3H), 1.83–1.58 (m, 2H), 1.56–1.23 (m, 4H). LRMS *m*/*z* (ES^–^) 188 [M − H]^–^. See Extended Data Fig. 2.

#### pIF-OH

A solution of 4-iodo-l-phenylalanine (2.0 g, 6.9 mmol, 1 equiv.) in AcOH (3 ml) and H_2_O (12 ml) was stirred at r.t. NaNO_2_ (2.4 g, 34.4 mmol, 5 equiv.) in H_2_O (7 ml) was then added to the amino acid solution dropwise over 10 min. The reaction was stirred at r.t. overnight. Then 1 M HCl (20 ml) was added and the mixture extracted with ethyl acetate (3 × 20 ml). The combined organic layers were dried over Na_2_SO_4_, filtered and concentrated. The crude hydroxy acid was purified by silica gel column chromatography eluting with ethyl acetate, hexane and acetic acid (50:48:2). The combined product fractions were concentrated to dryness to give pure pIF-OH (957 mg, 47% yield) as a colourless solid, LRMS *m*/*z* (ES^–^) 291 [M − H]^–^. See Extended Data Fig. 2.

#### NapA-OH

A solution of 3-(2-naphthyl)-l-alanine (215 mg, 1.0 mmol, 1 equiv.) in AcOH (1.8 ml) and H_2_O (3.4 ml) was stirred at r.t. NaNO_2_ (578 mg, 8.4 mmol, 8.4 equiv.) in H_2_O (2 ml) was then added to the amino acid solution dropwise over 5 min. The reaction was stirred at r.t. overnight. Then 1 M HCl (20 ml) was added and the mixture extracted with ethyl acetate (3 × 20 ml). The combined organic layers were dried over Na_2_SO_4_, filtered and concentrated. The crude hydroxy acid was purified by silica gel column chromatography eluting with ethyl acetate, hexane and acetic acid (50:48:2). The combined product fractions were concentrated to dryness to give pure NapA-OH (102 mg, 47% yield) as a colourless solid. ^1^H NMR analysis *δ*_H_ (400 MHz, CD_3_CN) 8.12–7.67 (m, 3H), 7.64–7.35 (m, 2H), 4.49 (dd, *J* = 7.9, 4.2 Hz, 1H), 3.29 (dd, *J* = 14.0, 4.3 Hz, 1H), 3.08 (dd, *J* = 14.0, 7.8 Hz, 1H). LRMS *m*/*z* (ES^–^) 215 [M − H]^–^. See Extended Data Fig. 2.

### Reporting summary

Further information on research design is available in the Nature Portfolio Reporting Summary linked to this article.

## Online content

Any methods, additional references, Nature Portfolio reporting summaries, source data, extended data, supplementary information, acknowledgements, peer review information; details of author contributions and competing interests; and statements of data and code availability are available at 10.1038/s41557-022-01082-0.

## Supplementary information

Supplementary Information Supplementary Figs. 1–10 and Table 1.

Reporting Summary

Supplementary Table 2 Plasmids and primers in this study.

Supplementary Data 1 Raw source data for Fig. 5c.

## Data Availability

Data supporting the findings of this study are included in the article and Supplementary Information. Alternatively, data is available from the corresponding authors upon reasonable request. Source data are provided with this paper.

## Source data

Source Data Fig. 2 Statistical source data.
Source Data Fig. 3 Statistical source data.
Source Data Fig. 4 Statistical source data.

## Code availability

The code for the automated orthogonal aaRS/tRNA pair combinations with orthogonal substrates is available at https://github.com/JWChin-Lab.
