# Supplementary material for: Genetically programmed cell-based synthesis of non-natural peptide and depsipeptide macrocycles
Source: Nat Chem. 2022 Dec 22;15(1):61–9. doi: 10.1038/s41557-022-01082-0 (PMC9836938; doi:10.1038/s41557-022-01082-0)

nature chemistry

Article https://doi.org/10.1038/s41557-022-01082-0

Genetically programmed cell-based synthesis of non-natural peptide and depsipeptide macrocycles

In the format provided by the authors and unedited

Supplementary Information

Genetically programmed cell-based synthesis of non-natural peptide and depsipeptide macrocycles

Martin Spinck[1,2], Carlos Piedrafita[1,2], Wesley E. Robertson[1,*] Thomas S. Elliott[1], Daniele Cervettini[1], Daniel de la Torre[1], Jason W. Chin[1,*]

[1]Medical Research Council Laboratory of Molecular Biology, Francis Crick Avenue, Cambridge, England, UK

[2]These authors contributed equally.

[*]Correspondence: chin@mrc-lmb.cam.ac.uk, wesr@mrc-lmb.cam.ac.uk

Supplementary Figures

a **Sansalvamide A**

b **YM-254890**

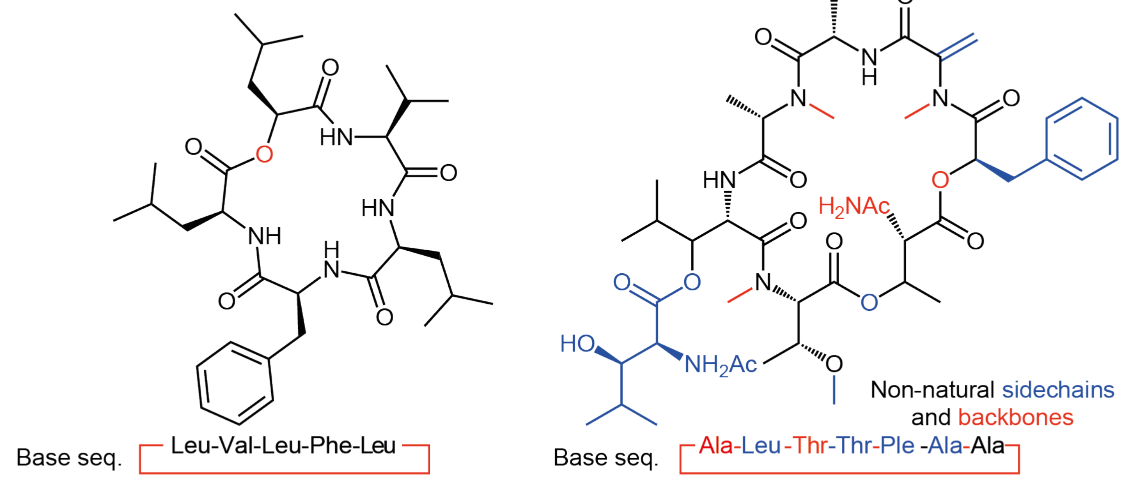

Supplementary Figure 1. Chemical structure of Sansalvamide A and YM-254890

a-b, Chemical structure Sansalvamide A (panel a) and YM-254890 (panel b) with modification of the backbone highlighted in red and non-natural sidechains coloured blue. The base sequence was derived from both peptides omitting all modifications.

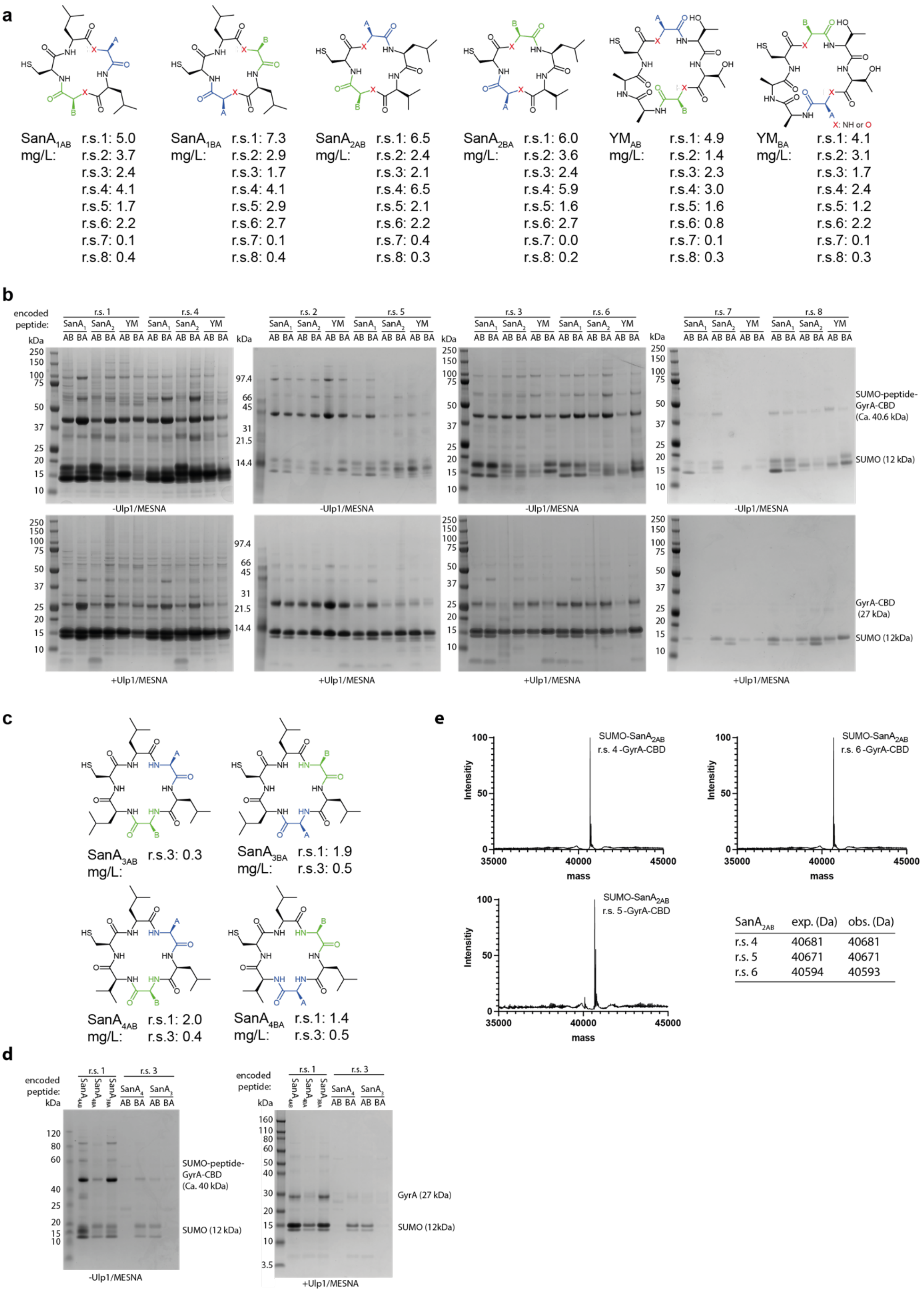
a
SanA1AB mg/L: r.s.1: 5.0 r.s.2: 3.7 r.s.3: 2.4 r.s.4: 4.1 r.s.5: 1.7 r.s.6: 2.2 r.s.7: 0.1 r.s.8: 0.4
SanA1BA mg/L: r.s.1: 7.3 r.s.2: 2.9 r.s.3: 1.7 r.s.4: 4.1 r.s.5: 2.9 r.s.6: 2.7 r.s.7: 0.1 r.s.8: 0.4
SanA2AB mg/L: r.s.1: 6.5 r.s.2: 2.4 r.s.3: 2.1 r.s.4: 6.5 r.s.5: 2.1 r.s.6: 2.2 r.s.7: 0.4 r.s.8: 0.3
SanA2BA mg/L: r.s.1: 6.0 r.s.2: 3.6 r.s.3: 2.4 r.s.4: 5.9 r.s.5: 1.6 r.s.6: 2.7 r.s.7: 0.0 r.s.8: 0.2
YMAB mg/L: r.s.1: 4.9 r.s.2: 1.4 r.s.3: 2.3 r.s.4: 3.0 r.s.5: 1.6 r.s.6: 0.8 r.s.7: 0.1 r.s.8: 0.3
YMBA mg/L: r.s.1: 4.1 r.s.2: 3.1 r.s.3: 1.7 r.s.4: 2.4 r.s.5: 1.2 r.s.6: 2.2 r.s.7: 0.1 r.s.8: 0.3
X: NH or O
b
encoded peptide:
-Ulp1/MESNA
+Ulp1/MESNA
SUMO-peptide-GyrA-CBD (Ca. 40.6 kDa)
SUMO (12 kDa)
GyrA-CBD (27 kDa)
SUMO (12kDa)
c
SanA3AB mg/L: r.s.3: 0.3
SanA3BA mg/L: r.s.1: 1.9 r.s.3: 0.5
SanA4AB mg/L: r.s.1: 2.0 r.s.3: 0.4
SanA4BA mg/L: r.s.1: 1.4 r.s.3: 0.5
d
encoded peptide:
-Ulp1/MESNA
+Ulp1/MESNA
SUMO-peptide-GyrA-CBD (Ca. 40 kDa)
SUMO (12 kDa)
GyrA (27 kDa)
SUMO (12kDa)
e
SUMO-SanA2AB r.s. 4 -GyrA-CBD
SUMO-SanA2AB r.s. 6 -GyrA-CBD
SUMO-SanA2AB r.s. 5 -GyrA-CBD
Intensity
mass
SanA2AB exp. (Da) obs. (Da)
r.s. 4 40681 40681
r.s. 5 40671 40671
r.s. 6 40594 40593

Supplementary Figure 2. SUMO-peptide-GyrA expressions and yields.

a, Yields of the Ni-NTA purified protein (mg/L) for all $SanA_1$, $SanA_2$ and YM sequences encoded in a His_6-SUMO-peptide-GyrA-CBD fusion in combination with all reassignment schemes (r.s. 1-8, from **Fig. 1, Fig. 5**).

b, SDS-PAGE analysis of the Ni^{2+}-NTA purified proteins for all reassignment schemes before and after treatment with Ulp1 protease and reductant (Ulp1/MESNA). Each lane contains 1% of the total purified protein or reaction mix. All samples were not heated to limit unspecific hydrolysis. The expected molecular weight for each of the bands before and after Ulp1/MESNA treatment is provided.

c, Yields of the Ni-NTA purified protein (mg/L) for the $SanA_3$ and $SanA_4$ sequences reported and corresponding SDS-PAGE gels of the purified protein before and after Ulp1/MESNA treatment.

d, SDS-PAGE analysis of the Ni^{2+}-NTA purified proteins for the $SanA_3$ and $SanA_4$ sequences with reassignment schemes 1 and 3 before and after treatment with Ulp1/MESNA. Each lane contains 1% of the total purified protein or reaction mix. All samples were not heated to limit unspecific hydrolysis. The expected molecular weight for each of the bands before and after Ulp1/MESNA treatment is provided.

e, ESI-MS of three representative purified SUMO-$SanA_{2AB}$-GyrA-CBD fusions using reassignment schemes 3-6. The expected (exp.) and observed (obs.) intact protein masses are shown.

a

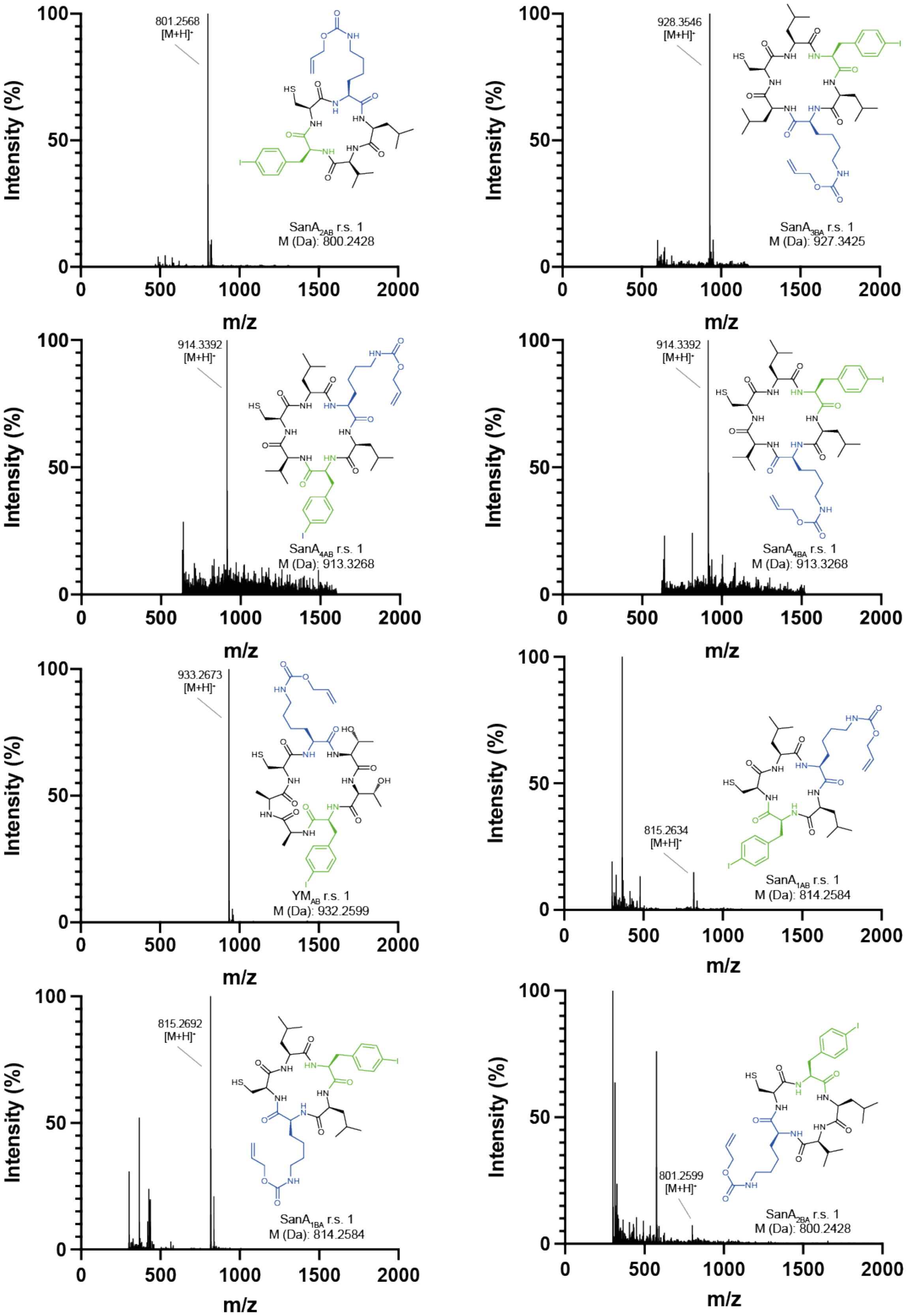
Intensity (%)
m/z
801.2568
[M+H]+
SanA2AB r.s. 1
M (Da): 800.2428
928.3546
[M+H]+
SanA3BA r.s. 1
M (Da): 927.3425
914.3392
[M+H]+
SanA4AB r.s. 1
M (Da): 913.3268
914.3392
[M+H]+
SanA4BA r.s. 1
M (Da): 913.3268
933.2673
[M+H]+
YMAB r.s. 1
M (Da): 932.2599
815.2634
[M+H]+
SanA1AB r.s. 1
M (Da): 814.2584
815.2692
[M+H]+
SanA1BA r.s. 1
M (Da): 814.2584
801.2599
[M+H]+
SanA2BA r.s. 1
M (Da): 800.2428

b

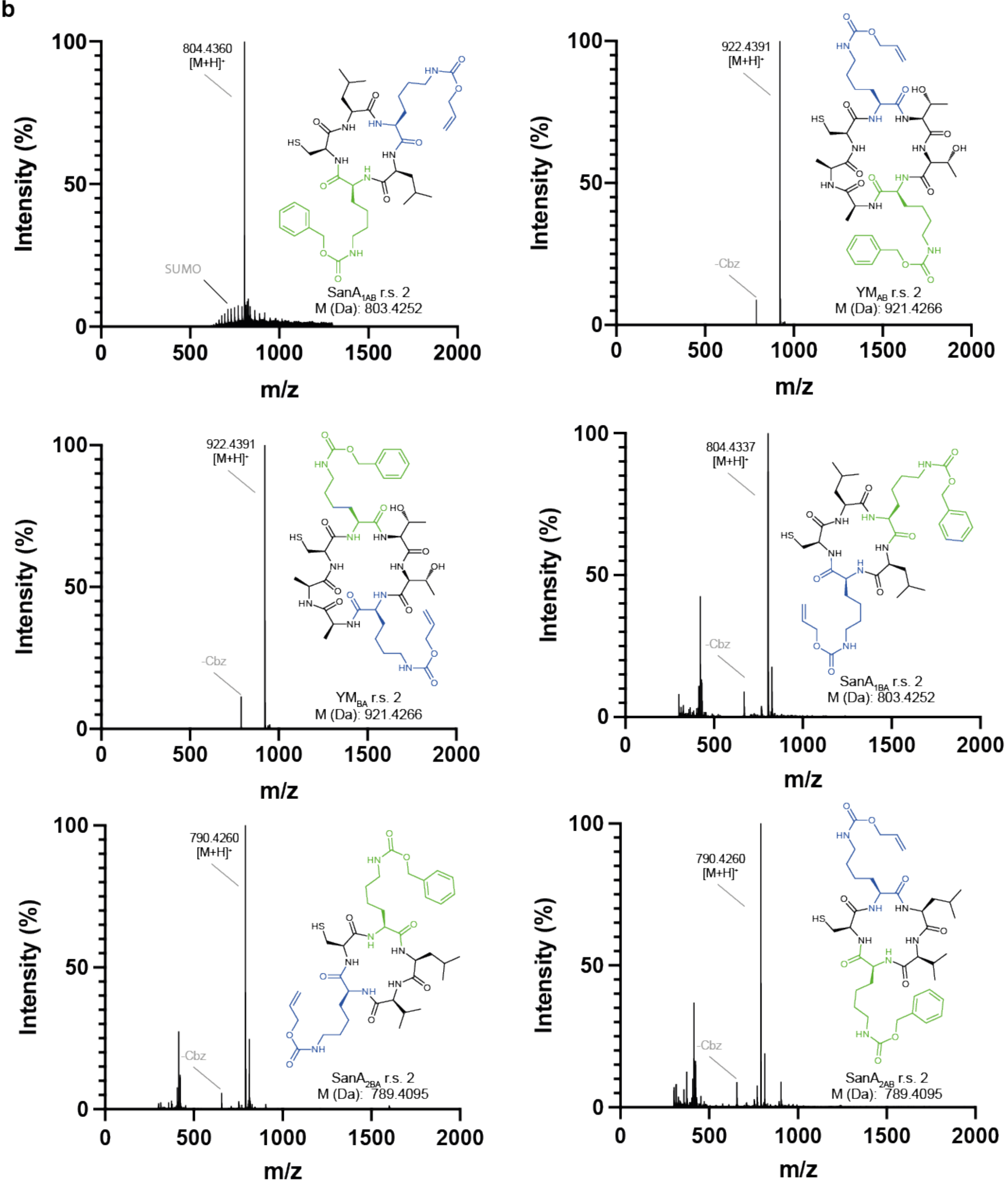

804.4360
[M+H]+
SUMO
SanA1AB r.s. 2
M (Da): 803.4252
922.4391
[M+H]+
-Cbz
YMAB r.s. 2
M (Da): 921.4266
922.4391
[M+H]+
-Cbz
YMBA r.s. 2
M (Da): 921.4266
804.4337
[M+H]+
-Cbz
SanA1BA r.s. 2
M (Da): 803.4252
790.4260
[M+H]+
-Cbz
SanA2BA r.s. 2
M (Da): 789.4095
790.4260
[M+H]+
-Cbz
SanA2AB r.s. 2
M (Da): 789.4095
Intensity (%)
m/z

c

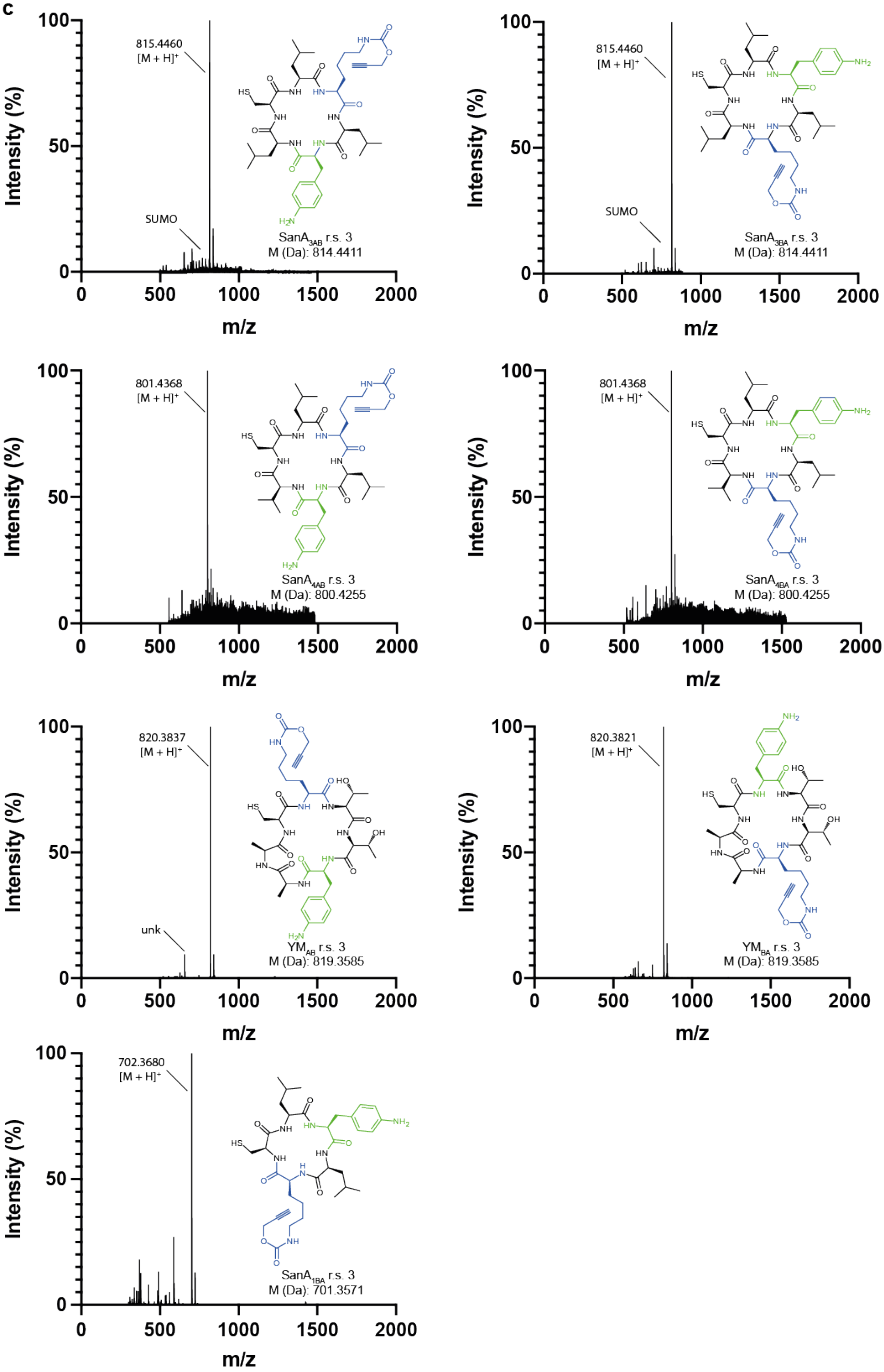

815.4460
[M + H]+
SUMO
SanA3AB r.s. 3
M (Da): 814.4411
815.4460
[M + H]+
SUMO
SanA3BA r.s. 3
M (Da): 814.4411
801.4368
[M + H]+
SanA4AB r.s. 3
M (Da): 800.4255
801.4368
[M + H]+
SanA4BA r.s. 3
M (Da): 800.4255
820.3837
[M + H]+
unk
YMAB r.s. 3
M (Da): 819.3585
820.3821
[M + H]+
YMBA r.s. 3
M (Da): 819.3585
702.3680
[M + H]+
SanA1BA r.s. 3
M (Da): 701.3571
Intensity (%)
m/z

c (continued)

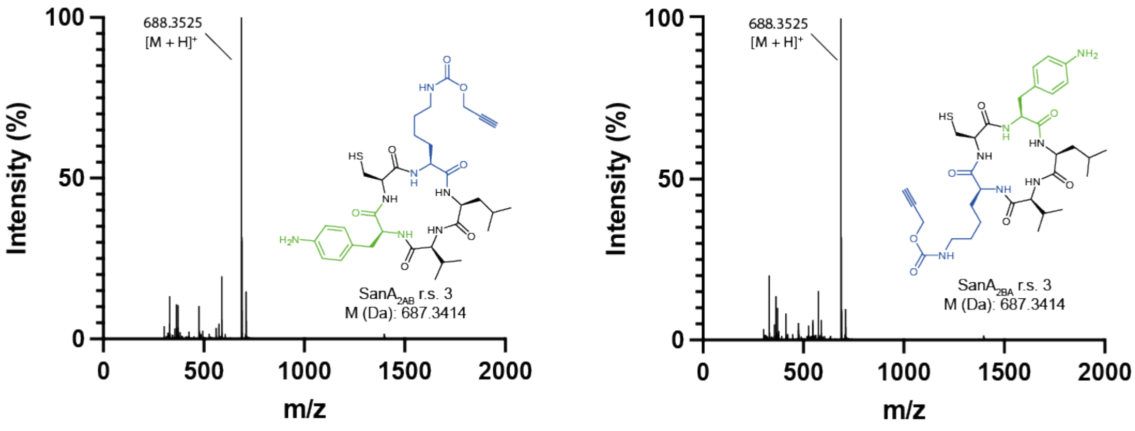

Supplementary Figure 3. ESI-MS spectra of the isolated cyclic peptides encoding the noncanonical amino acids defined in r.s. 1-3.

a-c, Liquid chromatography mass spectrometry (LC-MS) data for all successfully isolated cyclic peptides produced using r.s. 1 (panel a), r.s. 2 (panel b) or r.s. 3 (panel c), as shown in **Fig. 1,** monomer A is coloured in blue and monomer B in green. The chemical structure and expected mass for each peptide is shown and coloured according to **Fig. 1**. The product peak for each peptide is labeled and other related peaks relative to the product peak. Traces of SUMO protein remaining from the reaction were labeled if detected. The spectra of SanA$_2$ r.s. 2 is used as a reference in **Supplementary Fig. 9** and depicted there.

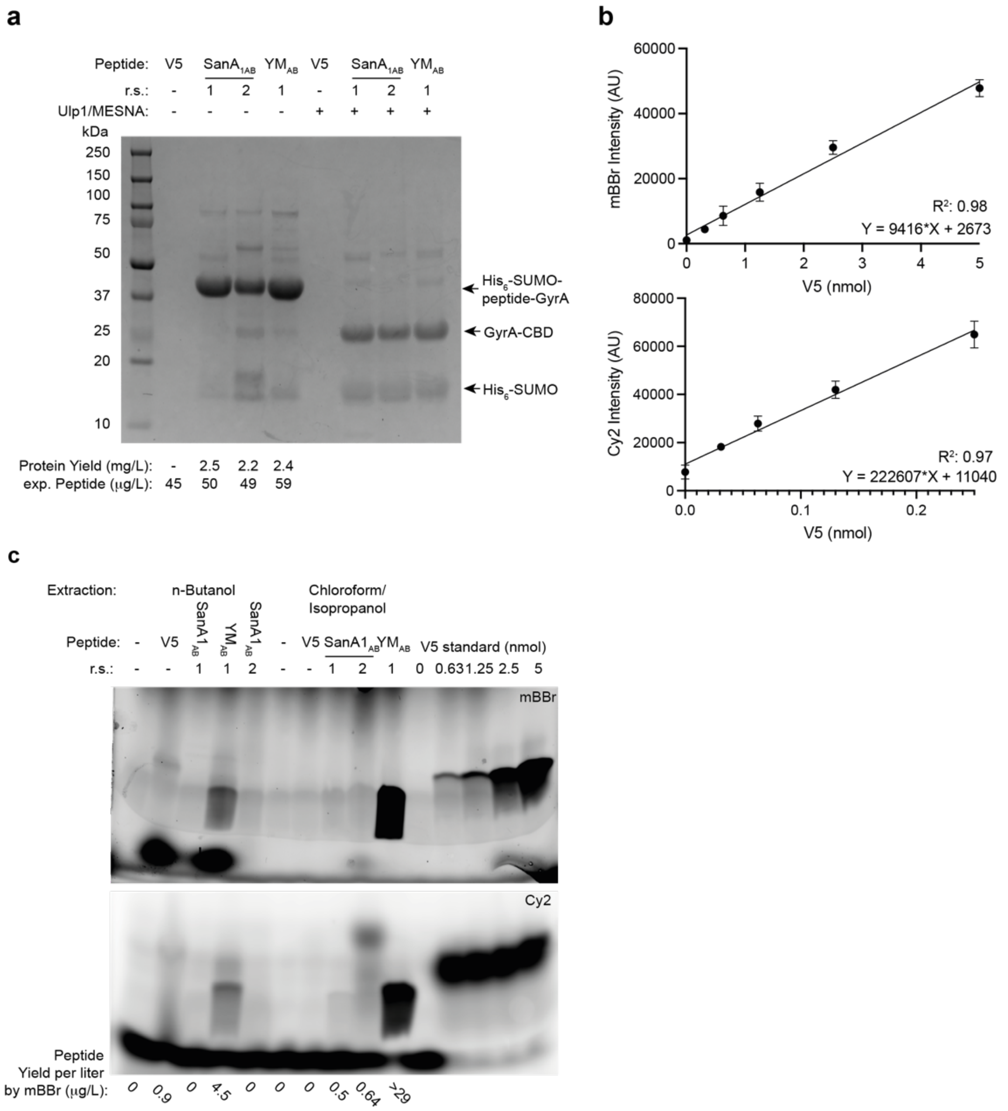

Supplementary Figure 4. Peptide production, extraction and quantification.

a, SDS-PAGE of purified His$_6$-SUMO-peptide-GyrA-CBD fusion proteins and their cleavage products. Each protein was purified from a 500 mL expression culture and eluted in 1 mL (1% of this elution was loaded). The ‘protein yield’ in mg per L of culture was measured and the expected amount of peptide (exp. Peptide) was calculated based on the molecular weight of the protein and each cyclic peptide. A control with 22.5 µg of pure V5 peptide (V5) in 1 mL reaction buffer was prepared (1525 Da, theoretically 45 µg/L), 1% of this control was loaded alongside the purified protein. All samples were treated with Ulp1 and reductant (Ulp1/MESNA, +), and 1% of the reaction after 18 h incubation was loaded. This resulted in

the near complete cleavage of His_6-SUMO-peptide-GyrA-CBD to His_6-SUMO and GyrA-CBD to release the peptide.

b, Fluorescence standard curve for monobromobimane (mBBr) and Alexa488-maleimide (Cy2 fluorescence) labelled V5 peptide; labelling reactions were performed in triplicate, run on SDS-PAGE, and the V5 peptide labelling with the fluorophore quantified using the Fiji, Gel-tools software.

c, Fluorescence imaging of an SDS-PAGE gel separating the labelled extracts. Each lane corresponds to peptides (V5 control, $SanA_{1AB}$ r.s.1, $SanA_{1AB}$ r.s.2, YM_{AB} r.s.1) extracted from 250 µL Ulp1/reductant reactions using n-Butanol or chloroform/isopropanol extraction (3:1). The extracts were labelled with monobromobimane (mBBr, +191 Da) or Alexa488-maleimide (Cy2, +718 Da). The amount of the extracted and labelled peptide was determined by quantifying the band intensity relative to the labelled V5 (mBBr) standards. An extracted yield in micrograms per L (µg/L) of the original protein expression culture was calculated to allow direct comparison with the expected yields in the cleavage reactions, and the known input concentration of the V5 peptide, both shown in panel **a**. The extraction yield of the V5 control peptide in n-butanol is 2%, and the V5 peptide is not extracted in chloroform/isopropanol. The extraction yield of YM_{AB} r.s.1 (932 Da) in chloroform/isopropanol is 49%, while the extraction yield of YM_{AB} r.s.1 in n-butanol is 8%. The extraction of $SanA_{1AB}$ r.s.1, $SanA_{1AB}$ r.s.2 is barely detectable even though the cleavage reactions (in panel **a**) indicate that similar amounts of these peptides and the YM_{AB} r.s.1 peptide are present in the Ulp1 cleavage reactions. We note that this method labels free thiols and does not discriminate between cyclic and linear peptides. These experiments clearly demonstrate that extracted peptide yield is a function of the peptide sequence and the solvent used for extraction, and does not reflect the quantities of peptide in the Ulp1 cleavage reaction.

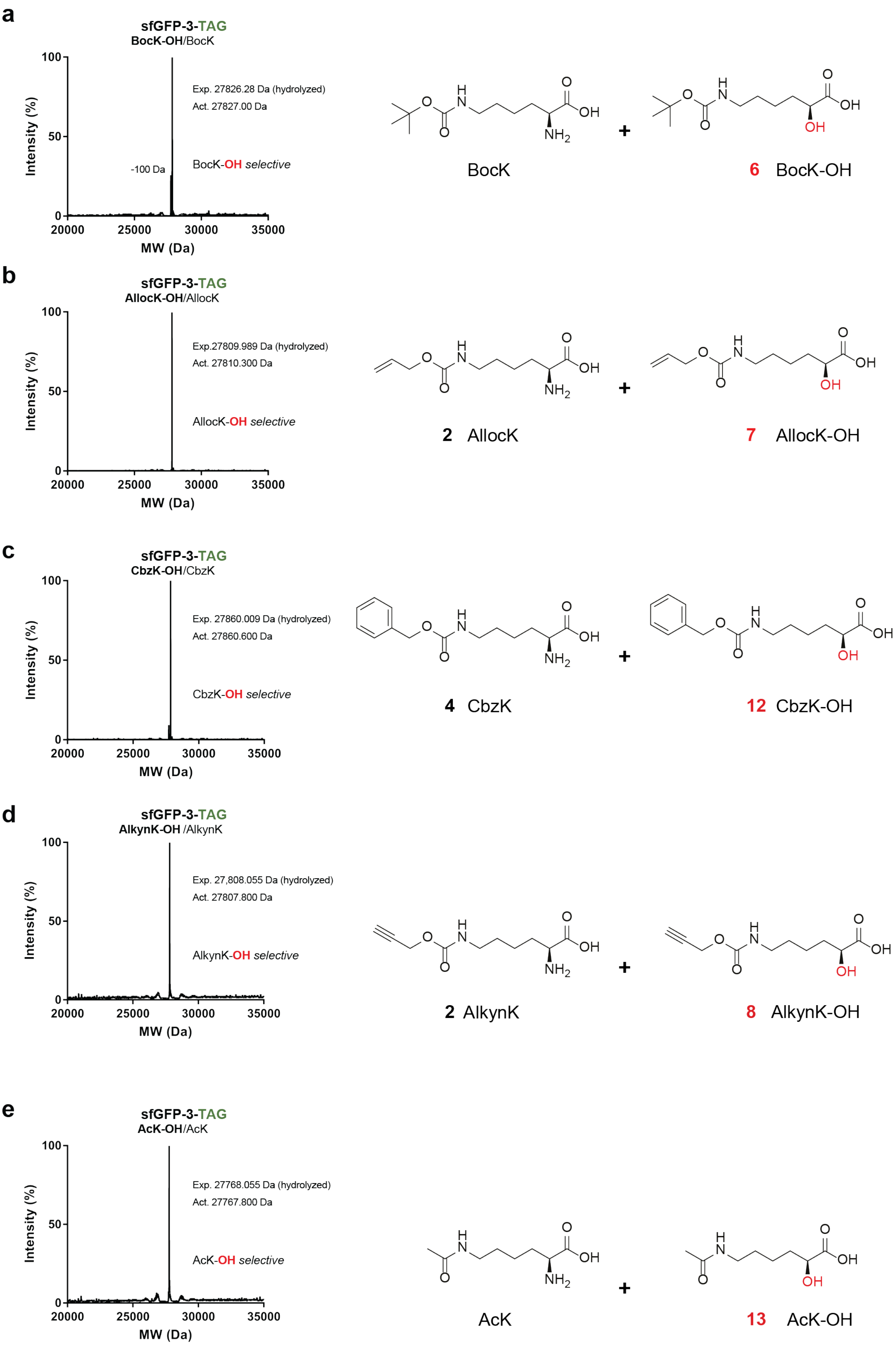
a
sfGFP-3-TAG
BocK-OH/BocK
Exp. 27826.28 Da (hydrolyzed)
Act. 27827.00 Da
-100 Da
BocK-OH selective
Intensity (%)
MW (Da)
BocK
6 BocK-OH
b
sfGFP-3-TAG
AllocK-OH/AllocK
Exp.27809.989 Da (hydrolyzed)
Act. 27810.300 Da
AllocK-OH selective
2 AllocK
7 AllocK-OH
c
sfGFP-3-TAG
CbzK-OH/CbzK
Exp. 27860.009 Da (hydrolyzed)
Act. 27860.600 Da
CbzK-OH selective
4 CbzK
12 CbzK-OH
d
sfGFP-3-TAG
AlkynK-OH /AlkynK
Exp. 27,808.055 Da (hydrolyzed)
Act. 27807.800 Da
AlkynK-OH selective
2 AlkynK
8 AlkynK-OH
e
sfGFP-3-TAG
AcK-OH/AcK
Exp. 27768.055 Da (hydrolyzed)
Act. 27767.800 Da
AcK-OH selective
AcK
13 AcK-OH

Supplementary Figure 5. Competition assay to measure the selectivity of pyrrolysyl-tRNA synthetases for hydroxy acids over amino acids.

a-e, The *M. mazei (Mm)* and *1R26* orthogonal pyrrolysyl-tRNA synthetase (PylRS)/tRNA pairs are selective for hydroxy acids over amino acids. In DH10B cells containing the *Mm*PylRS/*Mm*tRNA$^{Pyl}_{CUA}$ pair, sfGFP-3-TAG was expressed in the presence of 2 mM (7.5 mM in the case of AlkynK, 10 mM in the case of AcK) amino acid and 2 mM (10 mM in the case of AcK-OH) of its corresponding hydroxy acid analogue. When supplemented with BocK and BocK-OH (panel a), AllocK and AllocK-OH (panel b), CbzK and CbzK-OH (panel c), AlkynK and AlkynK-OH (panel d), or AcK and AcK-OH (panel e) electrospray ionization mass spectrometry (ESI-MS) analysis of Ni^{2+}-NTA purified sfGFP-3-TAG yielded a mass corresponding to the selective incorporation of the hydroxy acid over the amino acid. The -100 Da peak corresponds to loss of the *tert*-butoxycarbonyl in BocK. Analogously in cells containing the *1R26*PylRS(CbzK)/*Alv*tRNA$^{\Delta NPyl(8)}_{CUA}$ pair, ESI-MS analysis demonstrated the selectivity for CbzK-OH over CbzK (panel c).

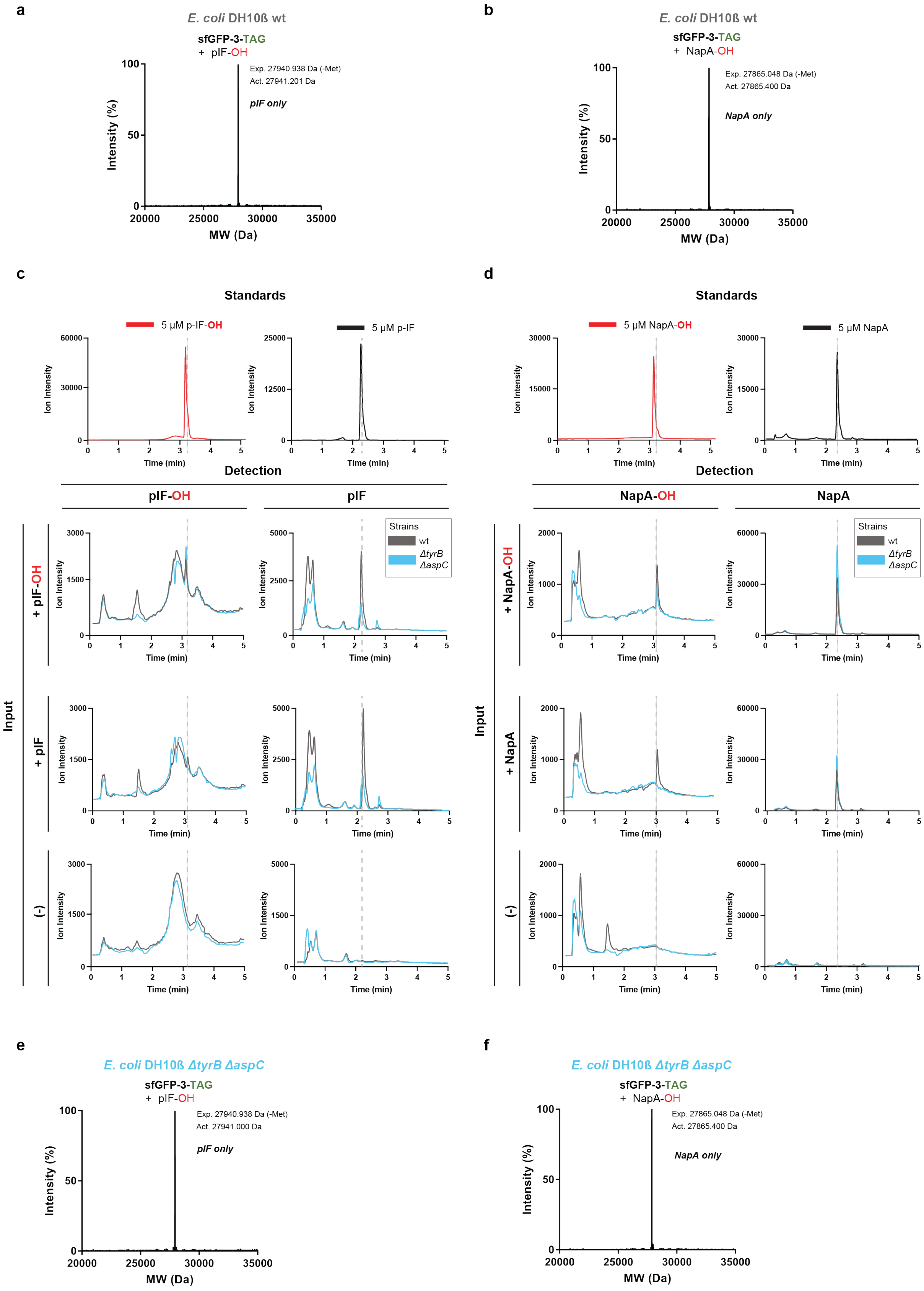

Supplementary Figure 6. Analysis of aromatic hydroxy acids intracellular concentrations and compatibility with orthogonal tyrosyl synthetase (TyrRS)/tRNA^{Tyr} pairs.

a-b, TyrRS variants incorporate amino acid analogues in DH10B wt cells supplemented with hydroxy acids. Purified sfGFP-3-TAG samples expressed with the *Af*TyrRS(pIF)/*Af*tRNA$^{Tyr}_{CUA}$ pair or the *Mj*TyrRS(Nap)/*Mj*tRNA$^{Tyr}_{CUA}$ pair in the presence of the hydroxy acids **15** (pIF-OH) or **16** (NapA-OH) were analyzed by LC-MS, revealing the intact mass corresponding to the incorporation of the cognate amino acid. *Af*TyrRS(pIF) is *Af*TyrRS-Y36I-L69M-H74L-Q116E-D165T-I166G[1], and *Mj*TyrRS(Nap) is *Mj*TyrRS-Y32L/D158P/I159A/L162Q/A167V[2].

c, Intracellular interconversion of **15** (pIF-OH) determined by liquid chromatography mass spectrometry (LC-MS). Hydroxy and amino acids were detected by an LC-MS assay with selected ion monitoring (SIM) mode and compared to 5 µM standards of each compound. *E. coli* DH10B strains (wt in gray, *ΔaspC/ΔtyrB* metabolic knockout in light blue) were grown in the presence of 1 mM pIF-OH (hydroxy acid, red traces) or 1 mM pIF (amino acid, grey traces) for 18 h, from which cell lysates were extracted and injected into the LC-MS.

d, Intracellular interconversion of **16** (Nap-OH) determined by liquid chromatography mass spectrometry (LC-MS). Experiments were performed essentially as described in panel a.

e-f, TyrRS variants incorporate amino acid analogues in DH10B *ΔaspC/ΔtyrB* metabolic knockout cells supplemented with hydroxy acids. Purified sfGFP-3-TAG samples expressed with the *Af*TyrRS(pIF)/*Af*tRNA$^{Tyr}_{CUA}$ pair or the *Mj*TyrRS(Nap)/*Mj*tRNA$^{Tyr}_{CUA}$ pair in the presence of the hydroxy acids **15** (pIF-OH) or **16** (NapA-OH) were analyzed by LC-MS, revealing the intact mass corresponding to the incorporation of the corresponding amino acids.

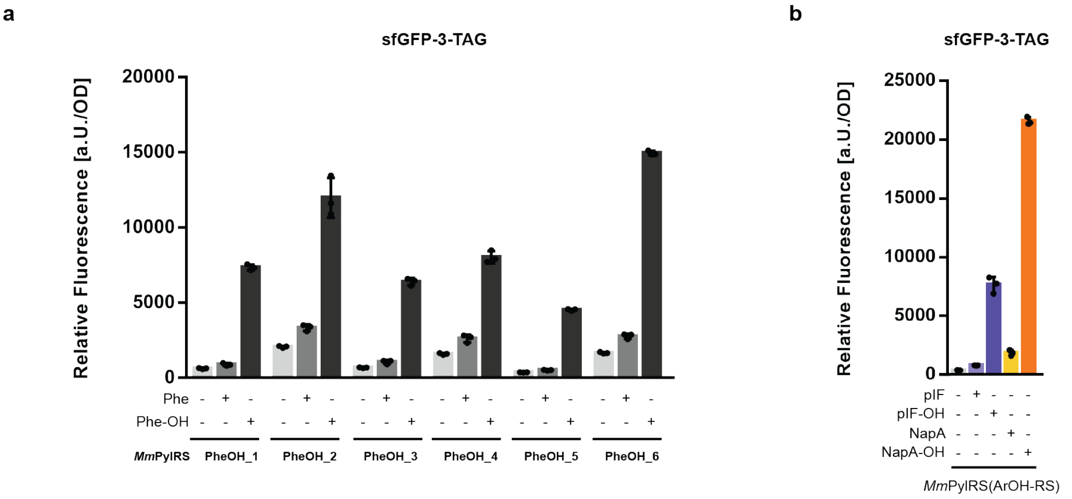

c

	Position	300	301	302	344	346	348	401	417
		Hydroxy group / Aromatic - side chain recognition					Expanded side chain recognition		
	*Mm*PylRS wt	M	L	A	M	N	C	V	W
*Mm*PylRS	PheOH_1	S	L	N	M	G	C	V	W
	PheOH_2	S	L	M	M	G	C	V	W
	PheOH_3	S	P	H	V	A	C	V	W
	PheOH_4	S	P	H	I	A	C	V	W
	PheOH_5	N	L	H	Q	V	C	V	W
	PheOH_6	S	L	H	L	A	C	V	W
	ArOH	S	L	H	L	A	S	L	T

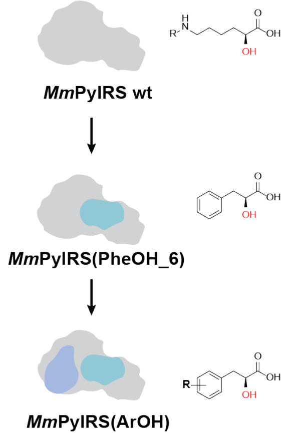

Supplementary Figure 7. Directed evolution of pyrrolysyl-tRNA synthetases that specifically incorporate hydroxy acids with aromatic side chains.

a, Specificity and activity of PheOH-RS variants. DH10B cells transformed with sfGFP-3-TAG and the respective PheOH-RS variant were grown in 2xYT media supplemented with 4 mM **14** phenyllactic acid (PheOH) or phenylalanine (Phe).

b, Specificity and activity of ArOH-RS. DH10B cells transformed with a sfGFP-3-TAG reporter and the respective PheOH-RS/*Mm*tRNA$^{Pyl}_{CUA}$ were grown in the presence of 2 mM **15** (pIF-OH) and **16** (NapA-OH) or 2 mM of their respective amino acid analogues, showing strong specificity for the hydroxy analogues.

c, Sequences of PheOH-RS and ArOH-RS variants. Based upon crystal structures of *Mm*PylRS, positions 300, 301, 302, 344, and 346 may be proximal to the hydroxy group and

phenyl ring recognition; these positions were targeted during the first round of evolution – this yielded PheOH-RS1-6. As part of the next evolution stage, we targeted positions 348, 401, and 417.

a

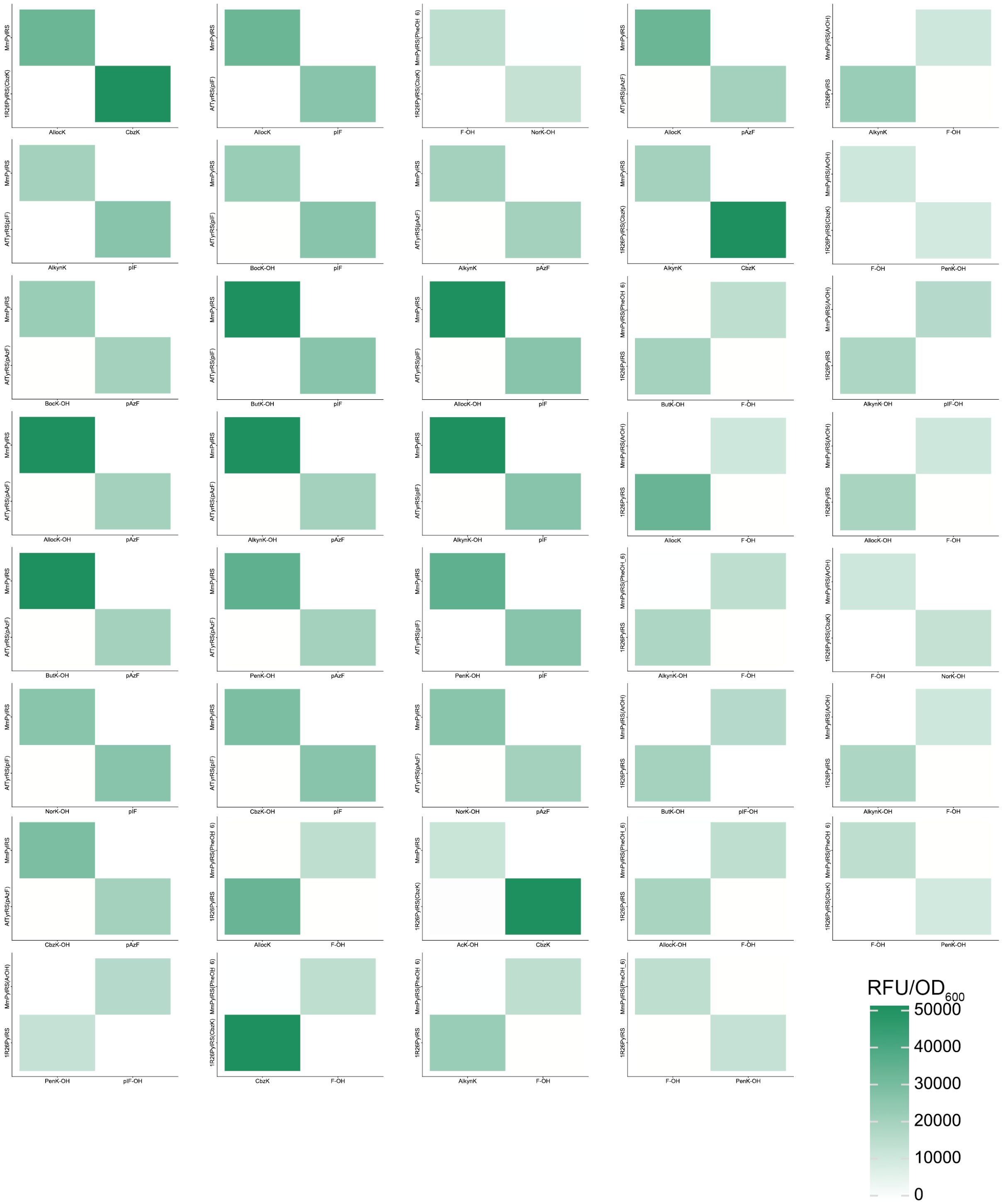
MmPyRS
1R26PyRS(CbzK)
AllocK
CbzK
AfTyrRS(pIF)
pIF
MmPyRS(PheOH_6)
F-OH
NorK-OH
AfTyrRS(pAzF)
pAzF
MmPyRS(ArOH)
1R26PyRS
AlkynK
PenK-OH
BocK-OH
ButK-OH
AllocK-OH
AlkynK-OH
pIF-OH
CbzK-OH
AcK-OH
RFU/OD600
50000
40000
30000
20000
10000
0

a *continued*

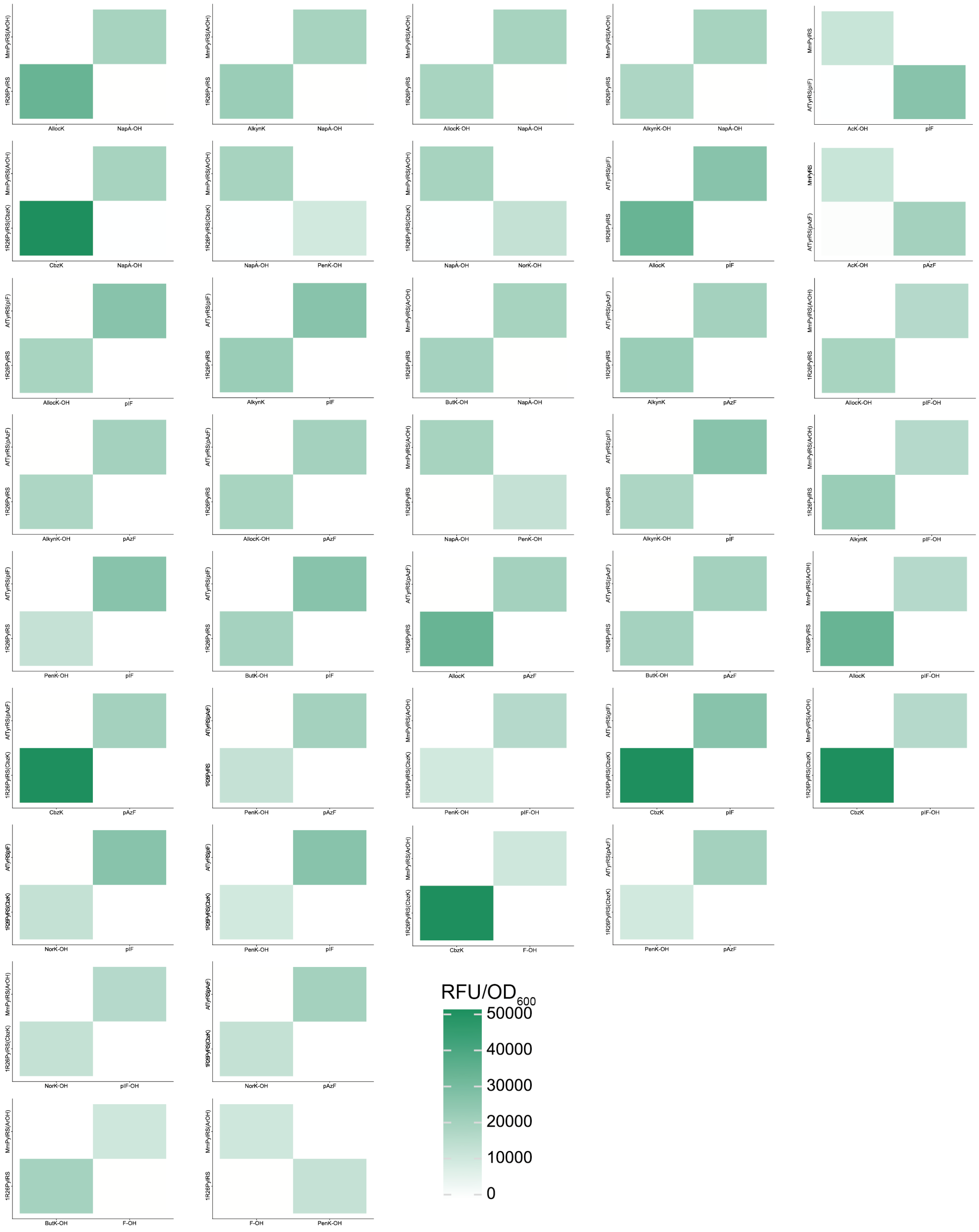
AllocK
NapA-OH
AlkynK
NapA-OH
AllocK-OH
NapA-OH
AlkynK-OH
NapA-OH
AcK-OH
pIF
CbzK
NapA-OH
NapA-OH
PenK-OH
NapA-OH
NorK-OH
AllocK
pIF
AcK-OH
pAzF
AllocK-OH
pIF
AlkynK
pIF
ButK-OH
NapA-OH
AlkynK
pAzF
AllocK-OH
pIF-OH
AlkynK-OH
pAzF
AllocK-OH
pAzF
NapA-OH
PenK-OH
AlkynK-OH
pIF
AlkynK
pIF-OH
PenK-OH
pIF
ButK-OH
pIF
AllocK
pAzF
ButK-OH
pAzF
AllocK
pIF-OH
CbzK
pAzF
PenK-OH
pAzF
PenK-OH
pIF-OH
CbzK
pIF
CbzK
pIF-OH
NorK-OH
pIF
PenK-OH
pIF
CbzK
F-OH
PenK-OH
pAzF
NorK-OH
pIF-OH
NorK-OH
pAzF
ButK-OH
F-OH
F-OH
PenK-OH
RFU/OD600
50000
40000
30000
20000
10000
0

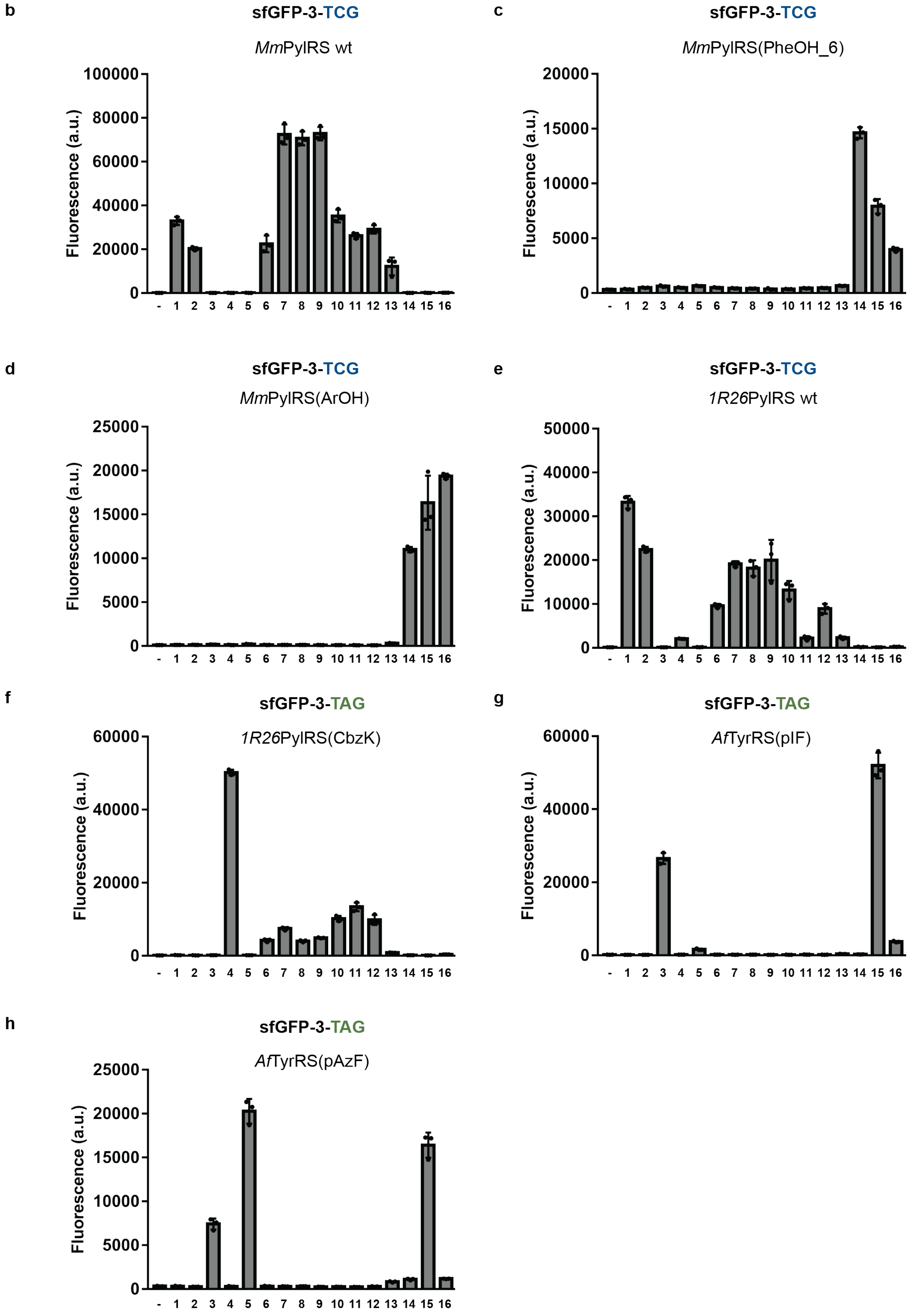
b
sfGFP-3-TCG
MmPylRS wt
Fluorescence (a.u.)
100000
80000
60000
40000
20000
0
- 1 2 3 4 5 6 7 8 9 10 11 12 13 14 15 16
c
sfGFP-3-TCG
MmPylRS(PheOH_6)
Fluorescence (a.u.)
20000
15000
10000
5000
0
- 1 2 3 4 5 6 7 8 9 10 11 12 13 14 15 16
d
sfGFP-3-TCG
MmPylRS(ArOH)
Fluorescence (a.u.)
25000
20000
15000
10000
5000
0
- 1 2 3 4 5 6 7 8 9 10 11 12 13 14 15 16
e
sfGFP-3-TCG
1R26PylRS wt
Fluorescence (a.u.)
50000
40000
30000
20000
10000
0
- 1 2 3 4 5 6 7 8 9 10 11 12 13 14 15 16
f
sfGFP-3-TAG
1R26PylRS(CbzK)
Fluorescence (a.u.)
60000
40000
20000
0
- 1 2 3 4 5 6 7 8 9 10 11 12 13 14 15 16
g
sfGFP-3-TAG
AfTyrRS(pIF)
Fluorescence (a.u.)
60000
40000
20000
0
- 1 2 3 4 5 6 7 8 9 10 11 12 13 14 15 16
h
sfGFP-3-TAG
AfTyrRS(pAzF)
Fluorescence (a.u.)
25000
20000
15000
10000
5000
0
- 1 2 3 4 5 6 7 8 9 10 11 12 13 14 15 16

Supplementary Figure 8. Identification of mutually orthogonal aaRS/tRNA pairs for encoding different combinations of two distinct non-canonical monomers.

a, We identified 77 mutually orthogonal aaRS/tRNA pairs which encode two distinct substrates, in which each pair recognizes a distinct substrate monomer. The absolute GFP florescence values (RFU/OD_{600}) for indicated aaRS/tRNA monomer combinations are shown, combinations with both cognate activities >10000 RFU/OD_{600} and both non-cognate activities < 1000 RFU/OD_{600} were considered a mutually orthogonal pair. From this analysis, we identified 77 pairs of mutually orthogonal aaRS/tRNA combinations which can encode 49 unique pairs of monomers; see also **Supplementary Table 1**.

b-h, Raw data (RFU/OD_{600}) bar graphs organized by aaRS active site variant. The raw data was used to generate the 2 x 2 orthogonality matrices in panel a and to generate the orthogonality matrix, following normalization, in **Fig. 4**.

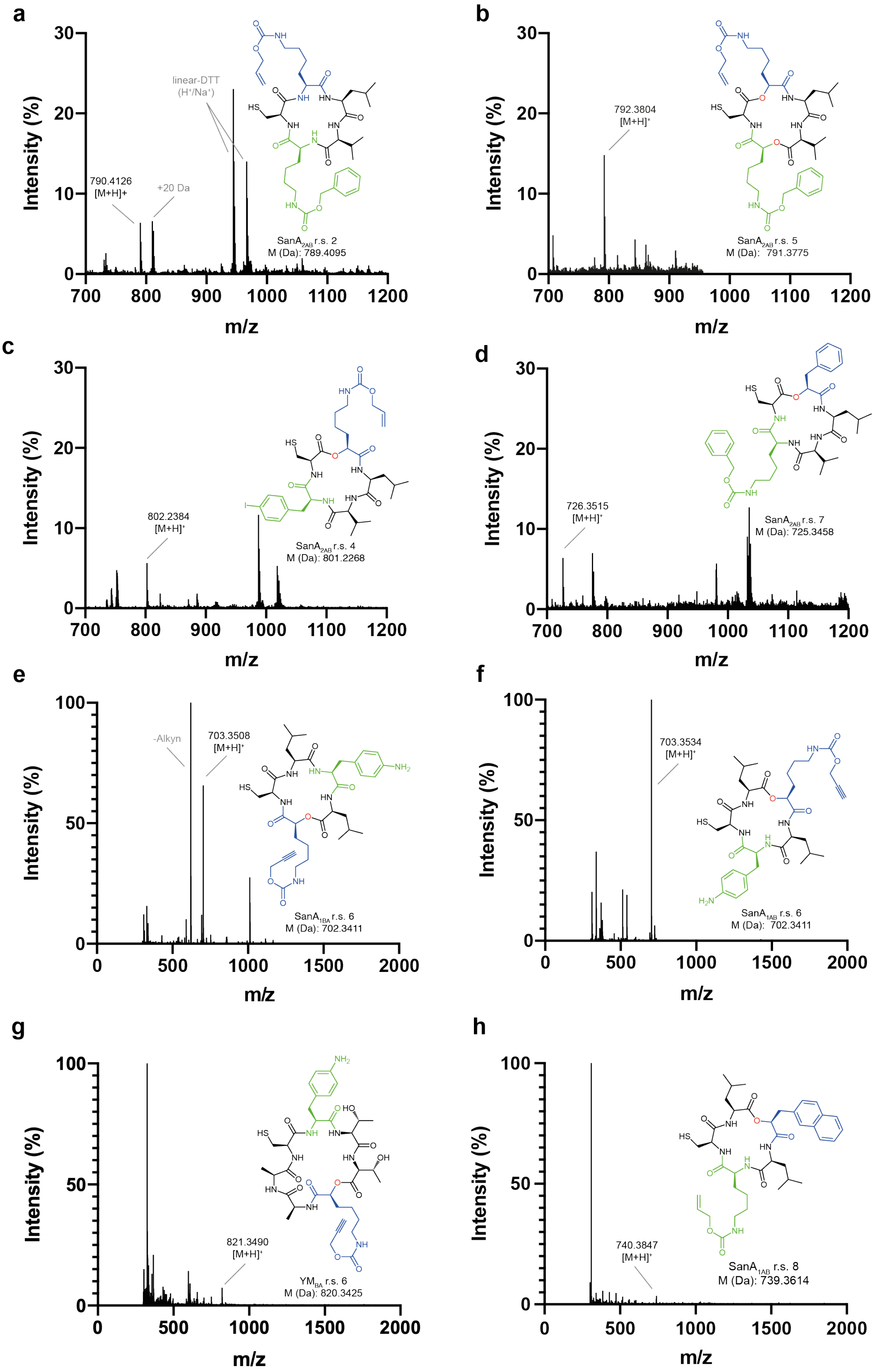

a
790.4126 [M+H]+
+20 Da
linear-DTT (H+/Na+)
SanA2AB r.s. 2
M (Da): 789.4095
b
792.3804 [M+H]+
SanA2AB r.s. 5
M (Da): 791.3775
c
802.2384 [M+H]+
SanA2AB r.s. 4
M (Da): 801.2268
d
726.3515 [M+H]+
SanA2AB r.s. 7
M (Da): 725.3458
e
-Alkyn
703.3508 [M+H]+
SanA1BA r.s. 6
M (Da): 702.3411
f
703.3534 [M+H]+
SanA1AB r.s. 6
M (Da): 702.3411
g
821.3490 [M+H]+
YMBA r.s. 6
M (Da): 820.3425
h
740.3847 [M+H]+
SanA1AB r.s. 8
M (Da): 739.3614
Intensity (%)
m/z

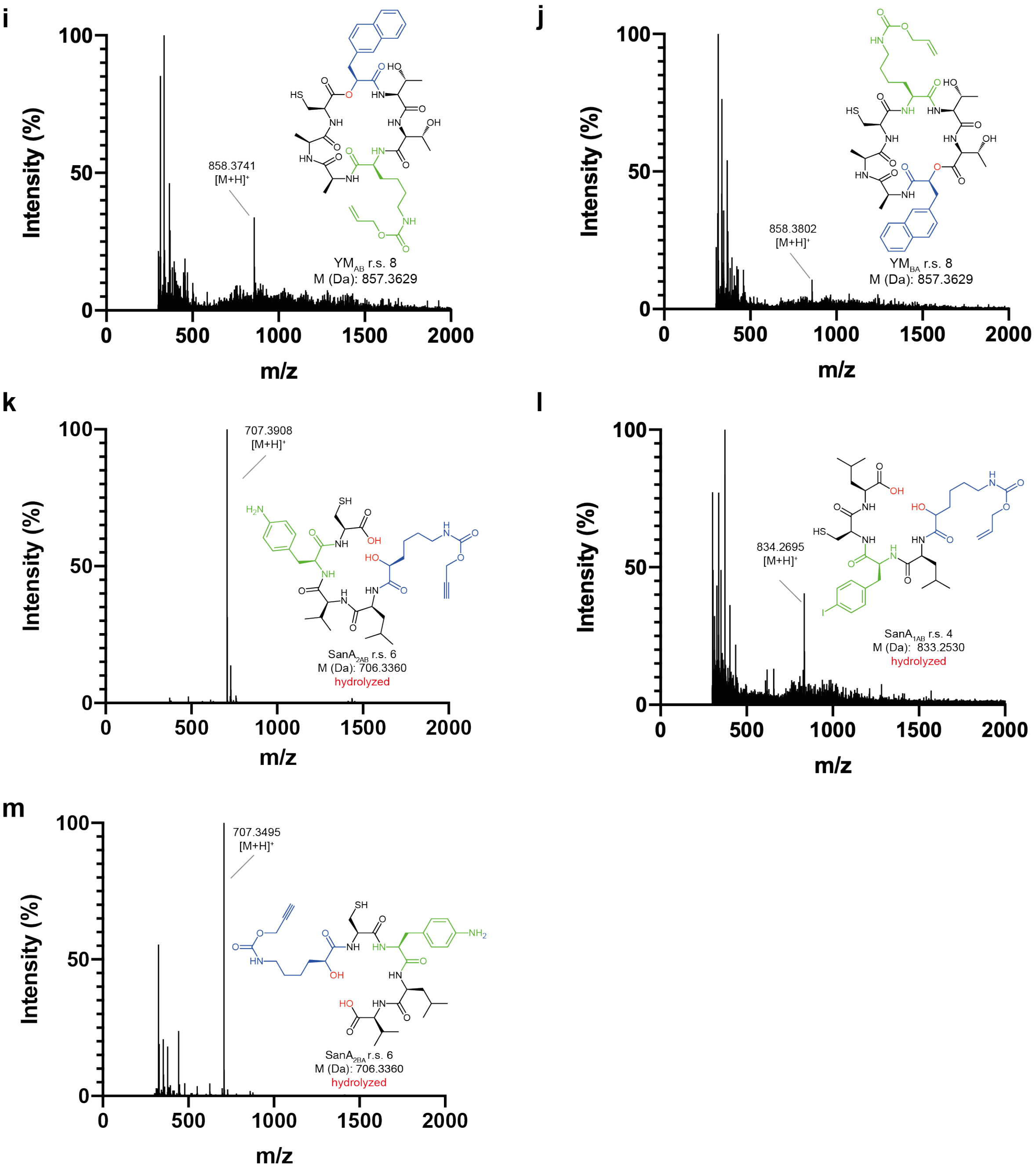

Supplementary Figure 9. Annotated spectra of cyclic depsipeptides and their hydrolysis products.

a-d, The chemical structures and the annotated LC-MS spectra for the depsipeptides listed in **Fig. 4b**. Monomer A is colored in blue and monomer B in green, the encoded ester bond is highlighted in red. Cyclic depsipeptides isolated as a fraction of hydrolyzed non-cyclized sequence, the hydrolyzed products are shown in **Supplementary Fig. 10**. For the $SanA_{2AB}$

sequence, the depsipeptide spectrum (r.s. 5, red) was overlaid with its corresponding amino analog (r.s. 2, black).

e-j, Annotated spectrum of all products observed as intact macrocyclic depsipeptide.

k-l, Depsipeptides observed only as hydrolyzed linear products after hydrolytic cleavage of the encoded ester bond.

m, Detected traces of the hydrolyzed cyclic peptide $SanA_{2BA}$ r.s. 6 from **Fig. 5c**.

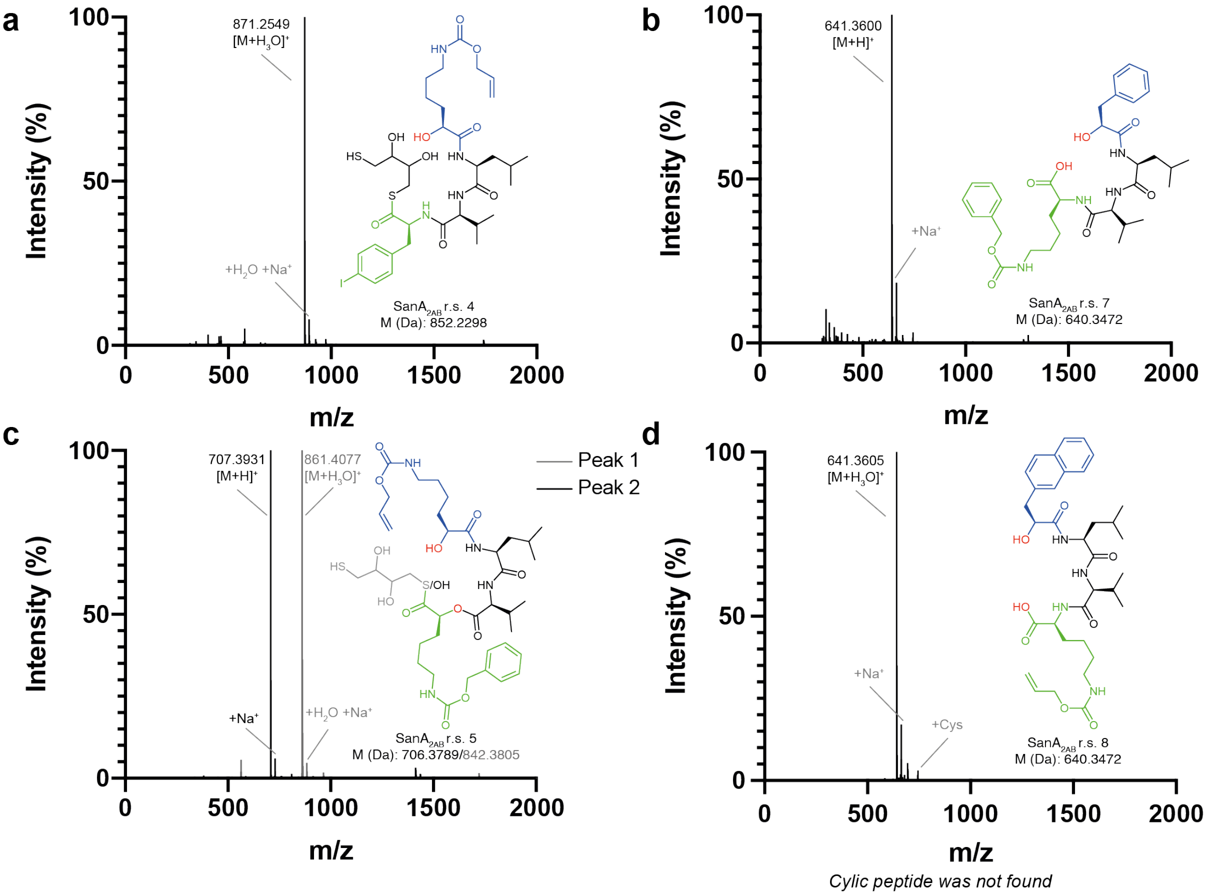

Supplementary Figure 10. Annotated mass spectra of isolated cyclic depsipeptides.

a-c, Structure and LC-MS spectra of the hydrolyzed linear products observed as by-product in the cyclisation attempts of the depsipeptides depicted in **Fig. 4**. Hydrolyzed products corresponding to the cyclic peptides in **Supplementary Fig. 9a-d**.

d, Hydrolyzed linear peptide of peptide SanA$_{2AB}$ r.s. 8; we did not observe the corresponding the cyclic peptide.

Supplementary Tables

All supplementary information tables are provided as separate Word or Excel files.

Supplementary Table 1

Annotated list of the identified mutually orthogonal aaRS/tRNA pairs for encoding different combinations of two distinct non-canonical monomers.

Supplementary Table 2

Annotated list of all plasmids and primers used in this study along with detailed sequence information of all aaRS gene nucleotide sequences.

Source Data S1

Raw data for sfGFP expression measurements from Fig. 2d.

Source Data S3

Raw data for sfGFP expression measurements from Fig. 3c.

Source Data S3

Raw data for sfGFP expression measurements from Fig.4a and SI Fig. 6c.

Source Data S4

Raw data for sfGFP expression measurements from SI Fig. 5.

References

1 Cervettini, D. *et al.* Rapid discovery and evolution of orthogonal aminoacyl-tRNA synthetase-tRNA pairs. *Nat Biotechnol* **38**, 989-999, doi:10.1038/s41587-020-0479-2 (2020).
2 Wang, L., Brock, A. & Schultz, P. G. Adding L-3-(2-Naphthyl) alanine to the genetic code of E. coli. *Journal of the American Chemical Society* **124**, 1836-1837 (2002).

Supplementary Table 1. Mutually orthogonal aaRS/tRNA pairs for encoding different combinations of two distinct non-canonical monomers. Some monomer combinations may be encoded by several aaRS/tRNA combinations with varying efficiency; all efficient combinations are listed. List derived from **Fig. 4**.

Monomers	aaRSs
AcK-OH - CbzK	MmPylRS - 1R26PylRS(CbzK)
AcK-OH - pAzF	MmPylRS - AfTyrRS(pAzF)
AcK-OH - pIF	MmPylRS - AfTyrRS(pIF)
AlkynK - CbzK	MmPylRS - 1R26PylRS(CbzK)
AlkynK - pAzF	MmPylRS - AfTyrRS(pAzF)<br>1R26PylRS - AfTyrRS(pAzF)
AlkynK - pIF	MmPylRS - AfTyrRS(pIF)<br>1R26PylRS - AfTyrRS(pIF)
AlkynK-OH - pAzF	MmPylRS - AfTyrRS(pAzF)<br>1R26PylRS - AfTyrRS(pAzF)
AlkynK-OH - pIF	MmPylRS - AfTyrRS(pIF)<br>1R26PylRS - AfTyrRS(pIF)
AllocK - CbzK	MmPylRS - 1R26PylRS(CbzK)
AllocK - pAzF	MmPylRS - AfTyrRS(pAzF)<br>1R26PylRS - AfTyrRS(pAzF)
AllocK - pIF	MmPylRS - AfTyrRS(pIF)<br>1R26PylRS - AfTyrRS(pIF)
AllocK-OH - pAzF	MmPylRS - AfTyrRS(pAzF)<br>1R26PylRS - AfTyrRS(pAzF)
AllocK-OH - pIF	MmPylRS - AfTyrRS(pIF)<br>1R26PylRS - AfTyrRS(pIF)
BocK-OH - pAzF	MmPylRS - AfTyrRS(pAzF)
BocK-OH - pIF	MmPylRS - AfTyrRS(pIF)
ButK-OH - pAzF	MmPylRS - AfTyrRS(pAzF)<br>1R26PylRS - AfTyrRS(pAzF)
ButK-OH - pIF	MmPylRS - AfTyrRS(pIF)<br>1R26PylRS - AfTyrRS(pIF)
CbzK - pAzF	1R26PylRS(CbzK) - AfTyrRS(pAzF)
CbzK - pIF	1R26PylRS(CbzK) - AfTyrRS(pIF)
CbzK-OH - pAzF	MmPylRS - AfTyrRS(pAzF)
CbzK-OH - pIF	MmPylRS - AfTyrRS(pIF)
F-OH - AlkynK	MmPylRS(PheOH_6) - 1R26PylRS<br>MmPylRS(ArOH) - 1R26PylRS
F-OH - AlkynK-OH	MmPylRS(PheOH_6) - 1R26PylRS<br>MmPylRS(ArOH) - 1R26PylRS
F-OH - AllocK	MmPylRS(PheOH_6) - 1R26PylRS<br>MmPylRS(ArOH) - 1R26PylRS
F-OH - AllocK-OH	MmPylRS(PheOH_6) - 1R26PylRS<br>MmPylRS(ArOH) - 1R26PylRS
F-OH - ButK-OH	MmPylRS(PheOH_6) - 1R26PylRS<br>MmPylRS(ArOH) - 1R26PylRS
F-OH - CbzK	MmPylRS(PheOH_6) - 1R26PylRS(CbzK)<br>MmPylRS(ArOH) -1R26PylRS(CbzK)

F-OH - NorK-OH	MmPylRS(PheOH_6) - 1R26PylRS(CbzK)<br>MmPylRS(ArOH) - 1R26PylRS(CbzK)
F-OH - PenK-OH	MmPylRS(PheOH_6) - 1R26PylRS,<br>MmPylRS(PheOH_6) - 1R26PylRS(CbzK)<br>MmPylRS(ArOH) - 1R26PylRS(CbzK)<br>MmPylRS(ArOH) - 1R26PylRS
NapA-OH - AlkynK	MmPylRS(ArOH) - 1R26PylRS
NapA-OH - AlkynK-OH	MmPylRS(ArOH) - 1R26PylRS
NapA-OH - AllocK	MmPylRS(ArOH) - 1R26PylRS
NapA-OH - AllocK-OH	MmPylRS(ArOH) - 1R26PylRS
NapA-OH - ButK-OH	MmPylRS(ArOH) - 1R26PylRS
NapA-OH - CbzK	MmPylRS(ArOH) - 1R26PylRS(CbzK)
NapA-OH - NorK-OH	MmPylRS(ArOH) - 1R26PylRS(CbzK)
NapA-OH - PenK-OH	MmPylRS(ArOH) - 1R26PylRS<br>MmPylRS(ArOH) - 1R26PylRS(CbzK)
NorK-OH - pAzF	MmPylRS - AfTyrRS(pAzF)<br>1R26PylRS (CbzK) - AfTyrRS(pAzF)
NorK-OH - pIF	MmPylRS - AfTyrRS(pIF)<br>1R26PylRS(CbzK) - AfTyrRS(pIF)
PenK-OH - pAzF	MmPylRS - AfTyrRS(pAzF)<br>1R26PylRS - AfTyrRS(pAzF)<br>1R26PylRS(CbzK) - AfTyrRS(pAzF)
PenK-OH - pIF	MmPylRS - AfTyrRS(pIF)<br>1R26PylRS - AfTyrRS(pIF)<br>1R26PylRS(CbzK) - AfTyrRS(pIF)
pIF-OH - AlkynK	MmPylRS(ArOH) - 1R26PylRS
pIF-OH - AlkynK-OH	MmPylRS(ArOH) - 1R26PylRS
pIF-OH - AllocK	MmPylRS(ArOH) - 1R26PylRS
pIF-OH - AllocK-OH	MmPylRS(ArOH) - 1R26PylRS
pIF-OH - ButK-OH	MmPylRS(ArOH) - 1R26PylRS
pIF-OH - CbzK	MmPylRS(ArOH) - 1R26PylRS(CbzK)
pIF-OH - NorK-OH	MmPylRS(ArOH) - 1R26PylRS(CbzK)
pIF-OH - PenK-OH	MmPylRS(ArOH) - 1R26PylRS,<br>MmPylRS(ArOH) - 1R26PylRS(CbzK)

SDS-Gels, SI Figure 2:

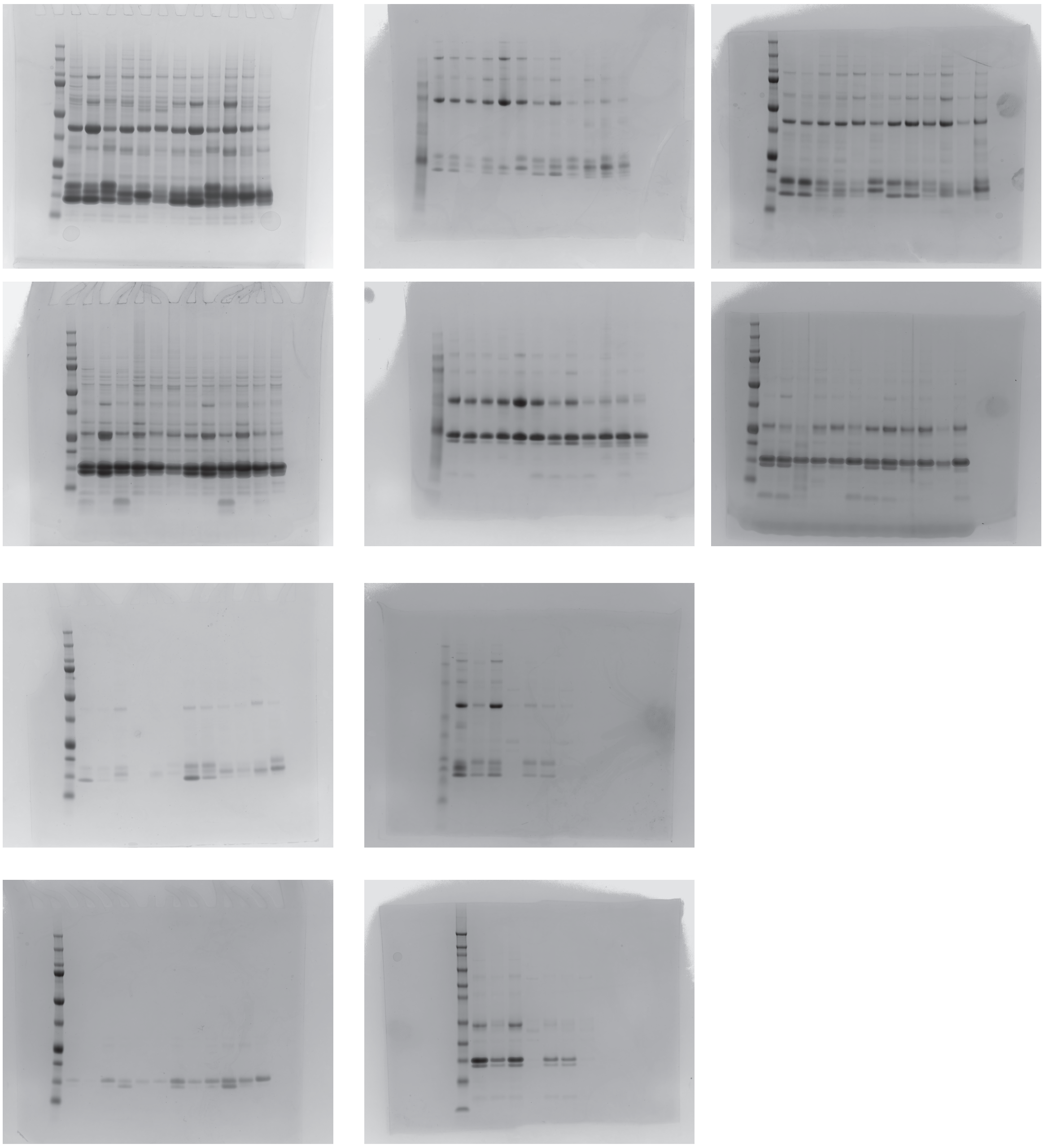

Gels, SI Figure 4:

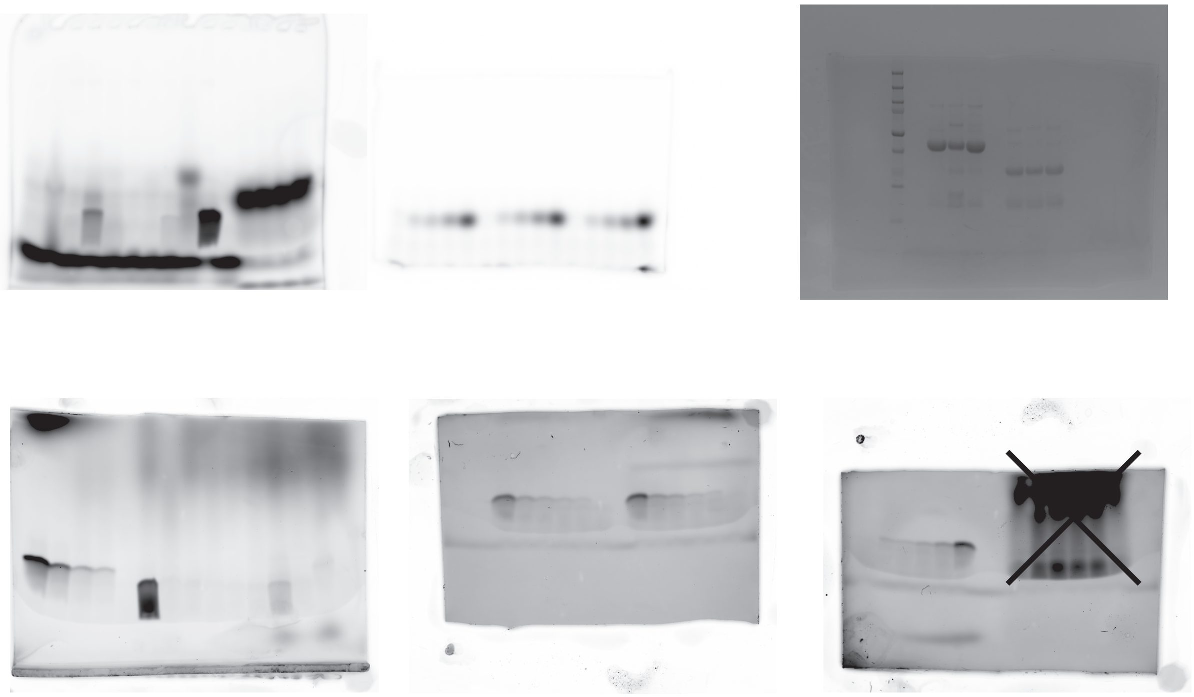

Supplement: Supplementary file 1 — Supplementary Figs. 1–10 and Table 1. [file 41557_2022_1082_MOESM1_ESM.pdf]
