# Supplementary material for: Genetically programmed cell-based synthesis of non-natural peptide and depsipeptide macrocycles
Source: Nat Chem. 2022 Dec 22;15(1):61–9. doi: 10.1038/s41557-022-01082-0 (PMC9836938; doi:10.1038/s41557-022-01082-0)
Supplement: Supplementary file 2 — Reporting Summary [file 41557_2022_1082_MOESM2_ESM.pdf]

nature portfolio

Corresponding author(s): Jason W. Chin

Last updated by author(s): Aug 25, 2022

Reporting Summary

Nature Portfolio wishes to improve the reproducibility of the work that we publish. This form provides structure for consistency and transparency in reporting. For further information on Nature Portfolio policies, see our Editorial Policies and the Editorial Policy Checklist.

Statistics

For all statistical analyses, confirm that the following items are present in the figure legend, table legend, main text, or Methods section.

n/a	Confirmed	
☐	☒	The exact sample size (n) for each experimental group/condition, given as a discrete number and unit of measurement
☐	☒	A statement on whether measurements were taken from distinct samples or whether the same sample was measured repeatedly
☒	☐	The statistical test(s) used AND whether they are one- or two-sided<br>*Only common tests should be described solely by name; describe more complex techniques in the Methods section.*
☐	☒	A description of all covariates tested
☐	☒	A description of any assumptions or corrections, such as tests of normality and adjustment for multiple comparisons
☐	☒	A full description of the statistical parameters including central tendency (e.g. means) or other basic estimates (e.g. regression coefficient) AND variation (e.g. standard deviation) or associated estimates of uncertainty (e.g. confidence intervals)
☒	☐	For null hypothesis testing, the test statistic (e.g. F, t, r) with confidence intervals, effect sizes, degrees of freedom and P value noted<br>*Give P values as exact values whenever suitable.*
☒	☐	For Bayesian analysis, information on the choice of priors and Markov chain Monte Carlo settings
☒	☐	For hierarchical and complex designs, identification of the appropriate level for tests and full reporting of outcomes
☒	☐	Estimates of effect sizes (e.g. Cohen's d, Pearson's r), indicating how they were calculated

Our web collection on statistics for biologists contains articles on many of the points above.

Software and code

Policy information about availability of computer code

Data collection	PheraSTAR plate reader used to collect sfGFP fluorescence and OD600 cell density measurements; raw data provided in Source Data S1. Waters Xevo G2 QTOF mass spectrometer used to collect mass spectra. Gel images were taken using a Typhoon (Cy2) or ChemiDoc imager (Ethidium bromide or Coomassie) using the default settings.
Data analysis	Graphpad Prism version 9 was used to generate all bar graphs and heat maps in this study. Waters MassLynx Mass Spectrometry Software was used to analyze mass spectra. Images were analyzed using Fiji Version 2.1.0/1.53c in-build tools for gel band quantification.

For manuscripts utilizing custom algorithms or software that are central to the research but not yet described in published literature, software must be made available to editors and reviewers. We strongly encourage code deposition in a community repository (e.g. GitHub). See the Nature Portfolio guidelines for submitting code & software for further information.

Data

Policy information about availability of data

All manuscripts must include a data availability statement. This statement should provide the following information, where applicable:

- Accession codes, unique identifiers, or web links for publicly available datasets
- A description of any restrictions on data availability
- For clinical datasets or third party data, please ensure that the statement adheres to our policy

All datasets and material generated or analysed in this study are available from the corresponding author upon reasonable request. Source data are provided with this paper.

Field-specific reporting

Please select the one below that is the best fit for your research. If you are not sure, read the appropriate sections before making your selection.

☒ Life sciences ☐ Behavioural & social sciences ☐ Ecological, evolutionary & environmental sciences

For a reference copy of the document with all sections, see nature.com/documents/nr-reporting-summary-flat.pdf

Life sciences study design

All studies must disclose on these points even when the disclosure is negative.

Sample size	No sample-size calculation was performed. Because the variation in the assays used is small and we are interested in large effects, the sample sizes used, as indicated in the manuscript, were deemed appropriate.
Data exclusions	There are no data exclusions.
Replication	The exact number of replicates is stated in the relevant figure legend. All attempts at replication were successful.
Randomization	No randomization. This is not relevant because the samples form defined groups.
Blinding	No blinding. Blinding is not relevant to the study. The groups were defined and studied by the investigator using standard protocols.

Reporting for specific materials, systems and methods

We require information from authors about some types of materials, experimental systems and methods used in many studies. Here, indicate whether each material, system or method listed is relevant to your study. If you are not sure if a list item applies to your research, read the appropriate section before selecting a response.

Materials & experimental systems

n/a	Involved in the study	
☒	☐	Antibodies
☒	☐	Eukaryotic cell lines
☒	☐	Palaeontology and archaeology
☒	☐	Animals and other organisms
☒	☐	Human research participants
☒	☐	Clinical data
☒	☐	Dual use research of concern

Methods

n/a	Involved in the study	
☒	☐	ChIP-seq
☒	☐	Flow cytometry
☒	☐	MRI-based neuroimaging
